# Major Shift of Influenza A Virus of Swine (IAV-S) by Human-to-Swine Spillover of the 2009 Pandemic Virus in Korea

**DOI:** 10.1155/2024/6366170

**Published:** 2024-08-24

**Authors:** Seung-Chai Kim, Taek Geun Lee, Eun-Jee Na, Sung-Hyun Moon, Hwan-Ju Kim, Chang-Gi Jeong, Young Ki Choi, Yeonsu Oh, Chung-Young Lee, Jae-Ku Oem, Won-Il Kim, Ho-Seong Cho

**Affiliations:** ^1^ College of Veterinary Medicine Jeonbuk National University, Iksan 54596, Republic of Korea; ^2^ College of Medicine and Medical Research Institute Chungbuk National University, Cheongju 28644, Republic of Korea; ^3^ Center for Study of Emerging and Re-Emerging Viruses Korea Virus Research Institute Institute for Basic Science (IBS), Daejeon 34126, Republic of Korea; ^4^ College of Veterinary Medicine and Institute of Veterinary Science Kangwon National University, Chuncheon 24341, Republic of Korea; ^5^ Department of Microbiology School of Medicine Kyungpook National University, Daegu 41566, Republic of Korea

## Abstract

The 2009 influenza A H1N1 pandemic (pdm09) originated from the influenza A virus of swine (IAV-S) through multiple reassortment events with avian and human IAVs. The pdm09 reportedly reintroduced the virus to pigs, contributing to the evolution and diversity of IAV-S through frequent reassortment and drifts. Surveillance and whole-genome sequencing of IAV-S from conventional pig farms in Korea during 2021–2022 revealed that the genetic diversity of H1 and H3 IAV-S was continuously enriched after human-to-swine spillover of pdm09 viruses with long-term maintenance, persistence, and reassortment of virus lineages. Evidence of additional human-to-swine spillover of viruses that are different from the 2009 virus but close to that of the recent H1N1pdm09 human vaccine was identified in this study. The identification of swine-adapted pdm09 viruses, which have accumulated amino acid mutations with potentially altered antigenicity and a unique potential N-glycosylation site within the haemagglutinin (HA) gene, suggests the distinctive evolution of spillover pdm09 viruses in swine. The genetic constellation of the recently emerging Eurasian avian-like swine lineage and the preexisting classical swine lineage H1 viruses in Korea has been expanded through reassortment with cocirculating pdm09 viruses and/or H3N2 IAV-S harboring the pdm09 M gene (H3N2pM). Collectively, after the major shift of Korean IAV-S from the classical swine lineage to the pdm09 lineage in 2009, the frequent spillover of pdm09 viruses and the circulation of IAV-S harboring pdm09 gene segments led to the continuous diversification of IAV-S through antigenic drift and shift, raising concerns about the potential reintroduction of these viruses to humans.

## 1. Introduction

Influenza A virus (IAV) is a zoonotic pathogen that remains an imminent global threat to public health and, even more so, to livestock welfare worldwide [1, 2]. Due to the segmented nature of their RNA genome and their error-prone RNA replication machinery, IAVs are highly genetically flexible, resulting in antigenically drifted viruses with a rapid evolution in surface proteins (antigenic drift) and rapid gene reassortment (antigenic shift), which occurs when two or more IAVs infect a cell or host and viral gene segments are reshuffled [1, 2, 3]. Surveillance of IAVs within the swine population is important because pigs are susceptible to avian, swine, and human IAVs and are considered “mixing vessels” in the generation of influenza viruses with pandemic potential [3, 4]. The first IAV pandemic of the twenty-first century, originating in swine in Mexico during March–May 2009 and killing approximately 575,000 people worldwide, was caused by a reassortant H1N1 virus termed “A(H1N1)pdm09” (pdm09) [5], which contained segments from avian, human, and swine viruses that had evolved in swine populations for over 90 years before virus emergence in humans [6, 7].

The H1N1, H3N2, and H1N2 subtypes of IAVs of swine (IAV-S) are endemic in pig populations worldwide, forming genetically distinct lineages of these subtypes that are related to their geographical distribution [8]. However, some IAV-S of North American and European lineages have been found frequently in Asia [8, 9], and the spread of the pdm09 virus in swine herds worldwide has generated an array of reassortments with various endemic IAV-S in Asia [10, 11], Europe [12, 13] and the Americas [14, 15]. Moreover, preexisting Eurasian avian-like (EA) H1N1 IAV-S in China since the early 2000s underwent reassortment with North American triple-reassortant (TR) IAV-S (H1N2 and pdm09) before and after the 2009 pandemic [16]. Some of these emergent EA reassortant IAV-S have caused human infections in China [17], and genotype 4 (G4) EA H1N1 viruses with high zoonotic potential have become predominant in China [18], posing potential threats of spreading to adjacent countries, including Korea. Thus, the circulation of IAV-S in Asia appears to be more complex than it is elsewhere, and continuous molecular surveillance of IAV-S is necessary.

In South Korea, H3N2 and H1N2 IAV-S were confirmed in 1998 and 2003, respectively, followed by a report of H1N1 in pigs in 2004 [19, 20]. Before the 2009 pandemic, IAV-S in Korea was believed to be genetically closely related to North American TR IAV-S, bearing a combination of human-like, classical swine-like, and avian-like segments, but evolved independently from their North American IAV-S counterparts, forming their own lineage in Korean swine herds [9, 21, 22]. However, since late 2009, pdm09 viruses with evidence of human-to-swine transmission were detected in the Korean pig population during a national survey [23, 24]. In addition, the pdm09 viruses have undergone reassortment with other IAV-S, resulting in the emergence of novel genotypes in the swine population, such as North American TR H3N2 IAV-S harboring a pdm09-like matrix (pM-like) segment (H3N2pM-like) [10, 25], which were predominantly found in North American swine and caused alarming human infections (termed “A(H3N2)v”), implying their potential threat to public health [26]. Reassortment of H1N1pdm09 viruses with preexisting Korean H1N2 IAV-S has also been reported, and these reassortant viruses not only were fully capable of infection and rapid transmission in pigs but also exhibited viral precursors with the ability to infect humans [11, 27]. Although the diversity of antigenically and genetically distinct IAV-S in the Korean swine population has been a potential concern for public health, recent epidemic knowledge of IAV-S in Korea is limited.

Therefore, we isolated IAV-S from nasal swabs collected from domestic swine from IAV-S-endangered pig farms from June 2021 to May 2022 in Korea. To update current IAV-S epidemiology in the Korean swine population and identify antigenic shifts or drifts that could lead to potential zoonotic threats, we performed a comprehensive phylogenetic analysis on human and swine H1N1, H1N2, and H3N2 sequence data collected between 2004 and 2022, including all strains that have been isolated in Korea. We found that the IAV-S detected in the 2021–2022 season, which were more genetically diverse than those in the past, were the result of independent evolution of pdm09 H1N1 viruses in the swine population, and continuous reassortments occurred between different IAV-S subtypes.

## 2. Materials and Methods

### 2.1. Sample Collection and IAV-S Detection

For active surveillance of IAV-S in South Korea, a total of 40 farms were selected from eight provinces (Gyeonggi-do, Chungcheongbuk-do, Chungcheongnam-do, Jeollabuk-do, Jeollanam-do, Gyeongsangbuk-do, Gyeongsangnam-do, and Jeju-do) except Gangwon-do province of South Korea with the similarly distributed numbers (4–6 farms). Field veterinarians at each province selected the farms with pigs showing respiratory clinical signs (IAV-S information unknown). From the same 40 farms, three nasal swabs and one pooled oral fluid sample from five different age groups (weaned, growing, finisher, sow, and gilt) as well as isolated sick pigs were collected for four seasons (720 nasal swabs and 240 pooled oral fluid samples for each season) from June 2021 to May 2022. Overall, a total of 2,880 nasal swabs and 960 oral fluid samples were submitted to Jeonbuk National University Veterinary Diagnostic Center (JBNU-VDC). RNA was extracted using the NP968 Nucleic Acid Extraction System (Xi'an Tianlong Science & Technology Co., Shaanxi, China). Conventional PCR for IAV-S detection was performed using the GenetBio SuPrimeScript RT-PCR kit according to the manufacturer's protocol. The following primers specific to the nucleoprotein of influenza A viruses as previously described with modifications were used: SIV-N-F: 5′-AAG CAG GGT AGA TAA TCA CTC A-3′; SIV-N-R: 5′-TAT GGG TCC TCC AGT TTT C-3′ [28].

### 2.2. Virus Isolation

PCR-positive samples were added to phosphate-buffered saline (PBS) containing 1% 100x antibiotic-antimycotic solution (Gibco, New York, USA) and centrifuged at 2,800x *g* for 10 min. The supernatants were filtered using a 0.45 *μ*m syringe filter (GVS, Sanford, ME, USA), and the filtered samples were inoculated into the allantoic cavities of specific-pathogen-free embryonated chicken eggs (9–11 days old) obtained from Soeng-Min Inc., Hwaseong, Korea. The eggs were incubated at 37°C for 72 hr and chilled at 4°C overnight. The allantoic fluids were collected and assessed for IAV-S using the haemagglutinin (HA) assay, following the guidelines of the World Organization for Animal Health (WOAH) standard [29]. When the HA assay result was negative in the allantoic fluids of the first passage, a second passage was subsequently conducted using the allantoic fluid from the first passage in eggs. If the allantoic fluid from the second passage was still negative for the HA assay, the sample was considered negative for SIV in this study [30]. The IAV-S in HA-positive samples was confirmed using primer sets targeting the M (pan-IAV), H1, H3, N1, and N2 genes, as previously described [31].

### 2.3. IAV-S Whole-Genome Sequencing

Viral RNA was extracted using a QIAamp® viral RNA Mini Kit (Qiagen Co., Hilden, Germany) following the manufacturer's instructions. Eight influenza RNA segments were amplified using multisegment RT-PCR with primers, as previously described [32]. With 0.1 *μ*M of each Uni12/Inf-1 (5′-GGGGGGAGCAAAAGCAGG-3′), Uni12/Inf-3 (5′-GGGGGGAGCGAAAGCAGG-3′) and Uni13/Inf-1 (5′-CGGGTTATTAGTAGAAAC AAGG-3′) primers, multiplex RT-PCR amplicons were generated using the SuperScriptTM III One-Step RT-PCR System with Platinum™ Taq High Fidelity DNA Polymerase (Thermo Fisher Scientific, Waltham, MA, United States) according to the manufacturer's instructions. The temperature cycle parameters were 42°C for 60 min, 94°C for 2 min, and then five cycles (94°C for 20 s, 44°C for 30 s, and 68°C for 3 min), followed by 31 cycles (94°C for 30 s, 57°C for 30 s, and 68°C for 7 min) with a final extension cycle of 68°C for 7 min. PCR products were purified using Agencourt AMPure XP (Beckman Coulter, Brea, CA, United States) according to the manufacturer's instructions.

Using the multiplex RT-PCR amplicons, the complete influenza virus genome was sequenced by the Illumina iSeq platform (Illumina, San Diego, CA, United States). Multiplexed sequencing libraries were prepared using the Nextera™ DNA Flex Library Prep Kit (Illumina) and Nextera™ DNA CD Indexes (Illumina). DNA libraries were analyzed using the Agilent 2100 Bioanalyzer (Agilent Technologies, Santa Clara, CA, United States) with an Agilent DNA 7500 Kit (Agilent Technologies). Sequencing was performed using a 300-cycle (150-bp paired-end) iSeq v2 reagent kit (Illumina) on the iSeq platform following the manufacturer's instructions. De novo assembly was performed with quality-trimmed total reads using CLC Genomics Workbench version v8.0. Acquired sequences were submitted to NCBI GenBank (accession number for HA segment; OR733507-OR733537, and other segments; OR762266-OR762482) (Table S3).

### 2.4. Data Collection

A total of 227 complete genome reference sequences, including Korean human IAVs (*n* = 48), global IAV-S (*n* = 126), global human IAVs (*n* = 42), and global avian IAVs (*n* = 11), were collected from the database of the National Center for Biotechnology Information (NCBI) (Bethesda, MD, USA) and the Global Initiative on Sharing Avian Influenza Data (GISAID) based on the IAV-S subclade nomenclature [33, 34]. To understand the phylogenetic relationship of the currently circulating Korean IAV-S, all available Korean IAV-S (*n* = 77) in NCBI GenBank or GISAID (collected in September 2022) and 30 IAV-S sequences obtained from this study were added to the dataset. Finally, a total of 334 complete genome sequences of IAVs were used for phylogenetic analysis (Tables S1 and S2).

### 2.5. Phylogenetic Analysis and Genotypic Diversity

Nucleotide sequences of the full gene of the viral PB2, PB1, PA, HA, NP, NA, M, and NS segments were aligned using Clustal Omega [35] and used to construct maximum-likelihood (ML) trees. The ML phylogenies of 304 sequences for each segment were reconstructed using RAxML-NG [36] with the general time-reversible nucleotide substitution model with a gamma distribution of among-site rate variation (GTR + G), and the reliability of the phylogenetic trees was evaluated with 1,000 bootstrap replications. Constructed ML trees were visualized and annotated using the *ggtree* package [37] in R [38]. We determined the genotypes of IAV-S by assigning each segment to specific lineages according to ML phylogenies and characterizing the genotype according to the clade distribution of its internal segments [39, 40].

To understand the evolutionary dynamics of IAV-S in Korea from 2004 to 2022, all 76 available H1 sequences and 31 available H3 sequences in Korea were further analyzed by the time-framed Bayesian inference method implemented on Bayesian evolutionary analysis by sampling trees (BEAST, v1.10.4) [41]. The GTR + G model of site rate variation using four rate category nucleotide substitutions and a lognormal uncorrelated relaxed clock model was selected for phylogenetic estimation. Four independent Markov chain Monte Carlo (MCMC) analyses were run for the H1, H3, N1, and N2 segments of Korean IAV-S for 50 million generations sampled every 5,000 runs, and the BEAGLE library was used to improve computational performance. Parameter convergence of the MCMC chains was assessed using Tracer (v1.7.1), and a minimum effective sample size (ESS) of 200 was needed. After the removal of 10% burn-in, a summary maximum clade credibility (MCC) tree was prepared using TreeAnnotator (v1.10.4) and visualized using ggtree [37].

### 2.6. Analysis of Amino Acid Mutations of A(H1N1)pdm09 Clades

The amino acid substitutions in the HA gene and NA gene of the pdm09 lineage were investigated, as divergent evolution into the human clade and swine clade was detected in the phylogenetic analysis. Multiple sequence alignment (MSA) of the amino acid sequences of all Korean pdm09 lineage sequences was generated using Clustal Omega [35], and identical sequences were eliminated. Amino acid (aa) mutations of H1N1 HA were then analyzed using the HA sequences of the A/California/07/2009 vaccine strain as a reference for the pdm09 lineage and the World Health Organization (WHO)-recommended vaccine strain A/Michigan/45/2015 as the second reference for the human clade. The potential N-linked glycosylation sites of each virus were predicted using NetNGlyc server 1.0 [42]. A three-dimensional structure map of pdm09 HA was reconstructed based on A/California/04/2009 (Protein Data Bank ID: 3AL4) using PyMOL Molecular Graphics System v4.6.0 [43], and the corresponding amino acid mutations and changes in N-linked glycosylation sites for each clade were visualized.

Additional antigenic characterization was performed using the sequence-based antigenic cartography method [44, 45], which enables the estimation of the antigenic distances (ADs) between H1 viruses based on amino acid differences in the five antigenic epitopes of HA. Amino acids in the five major epitopes (H1pdm numbering [46], Sa: 124, 125, 153–157, 159–164; Sb: 184–195; Ca1: 166–170, 203, 204, 205, 235–237; Ca2: 137–142, 221, 222; Cb: 70–75) were extracted manually, and an AD matrix was constructed by calculating and averaging the five epitopic distances (EDs) between each virus with the Hamming distance calculation utilizing the cultevo package in R software. Antigenic maps were generated by using the classical multidimensional scaling (MDS) with the stats package in R, applying a dimensional reduction to the AD matrix. Potential antigenic clusters were inferred based on the AD values observed between the clades and the clustering pattern analyzed by hierarchical clustering using the stats package in R. The calculated ADs were expressed in antigenic units (AU), which are linearly correlated with the gold-standard haemagglutination inhibition (HI) assay and indicate the existence of potential overlap recognition by antibodies at ADs < 8.0 AU [44, 45].

### 2.7. Hemagglutination Inhibition Assay

Hemagglutination inhibition (HI) assays were performed as previously described [29]. Briefly, all sera were treated with a receptor-disrupting enzyme (RDE) (Seiken, Japan) in a 1 : 1 ratio and incubated at 37°C for 18 hr, followed by heating at 56°C for 30 min to inactivate the RDE. The serial twofold dilutions of the treated sera were incubated with equal volumes of eight viral hemagglutinating units (HAU) of each virus for 30 min at room temperature. Then 1% chicken red blood cells were added, and hemagglutination activity was observed after 40 min of incubation. The HI titers were determined as the highest serum dilution that completely inhibited hemagglutination.

Ferret antisera established against A/California/07/09 (WHO-recommended pdm09 vaccine strain) and A/Busan/383/2017 (NCCP76097; representing A/Michigan/45/2015 which is the WHO-recommended pdm09 vaccine strain for the 2017/2018 season) were provided by the Korea Virus Research Institute (KVRI, https://www.ibs.re.kr/kvri/) of the Institute for Basic Science (IBS) and by the National Culture Collection for Pathogens (NCCP, https://nccp.kdca.go.kr/eng/main.do), National Institute of Infectious Diseases, National Institute of Health, respectively.

For comparison of antigenic properties, propagated viruses of human pdm09 strains A/Korea/01/2009 (NCCP43001) and A/Hawaii/70/2019 (NCCP43400; the WHO-recommended pdm09 vaccine strain for the 2020/2021 season) were also provided by the NCCP, Republic of Korea. Due to the unavailability of obtaining A/Michigan/45/2015 virus or antiserum, A/Hawaii/70/2019 virus and the antiserum against A/Busan/383/2017 were used as substitutes for A/Michigan/45/2015.

A total of eight viruses were selected for the HI assay, representing IAV-S clades circulating in Korea: early pdm09 human strain (A/Korea/01/2009), pdm09 human clade strain (A/Hawaii/70/2019), pdm09 human clade swine strain (Human-to-swine spillover; A/swine/Korea/0171/2022), pdm09 swine clade I (A/swine/Korea/0139/2022), pdm09 swine clade II (A/swine/Korea/0878/2021), Korean Classical Swine (CS) 1A.3.3.3 H1N2 clade (A/swine/Korea/0121/2022), and Korean Eurasian Avian-like Swine (EAS) 1C.2.3 H1N2 clade (A/swine/Korea/0608/2021).

## 3. Results

### 3.1. IAV-S Detection in 2021–2022

The collected specimens were screened by conventional RT-PCR without subtyping. There were 119 influenza-positive nasal swab samples (119/2,880, 4.1%) and 118 influenza-positive pooled oral fluid samples (118/960, 12.3%) among the 2,880 nasal swab and 960 pooled oral fluid samples tested, respectively. The peak IAV-S positive rate (6.4% of nasal swabs and 18.3% of pooled oral fluid) during the sampling period occurred during the winter season of 2021 (Table 1). The pooled oral fluid samples had a higher positive rate than the nasal swab specimens at both the sample level and the farm level. Twenty-eight viruses from 119 influenza-positive nasal swab samples and two viruses from 118 oral fluid samples were isolated, with virus isolation rates of 1.0% and 0.2%, respectively, from total samples, and isolation rates of 23.5% and 1.7%, respectively, from PCR-positive samples.

### 3.2. Lineage Classification

**Figure 1:**
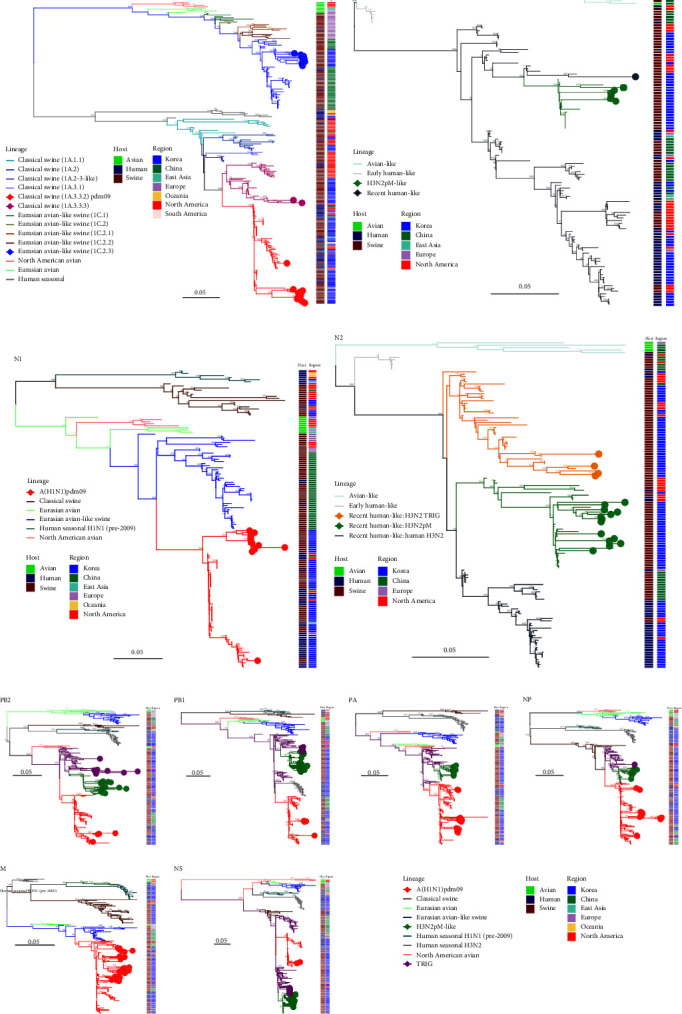
Maximum-likelihood (ML) phylogenetic trees and lineage classification of gene segments: HA-H1 (a), HA-H3 (b), NA-N1 (c), NA-N2 (d), and internal gene segments (e). Branches are colored by lineage of origin, and colored branch tips indicate strains isolated in this study, while reference strains are annotated with gray-colored tips. The tip shapes of triangles (human) and circles (swine) indicate the isolated host.

ML phylogenetic analysis of whole segments of 334 IAV-S was conducted, and the lineages of their surface proteins and internal genes were classified (Figures 1 and S2). Of note, among the internal genes, all H1N1, H1N2, and H3N2 IAV-S strains isolated in this study possessed the pdm09 M segment (Figure 1(e)), which could be associated with promoting transmissibility, as described in a guinea pig model [47].

### 3.3. Phylogenetic Relationships of the Glycoproteins

**Figure 2:**
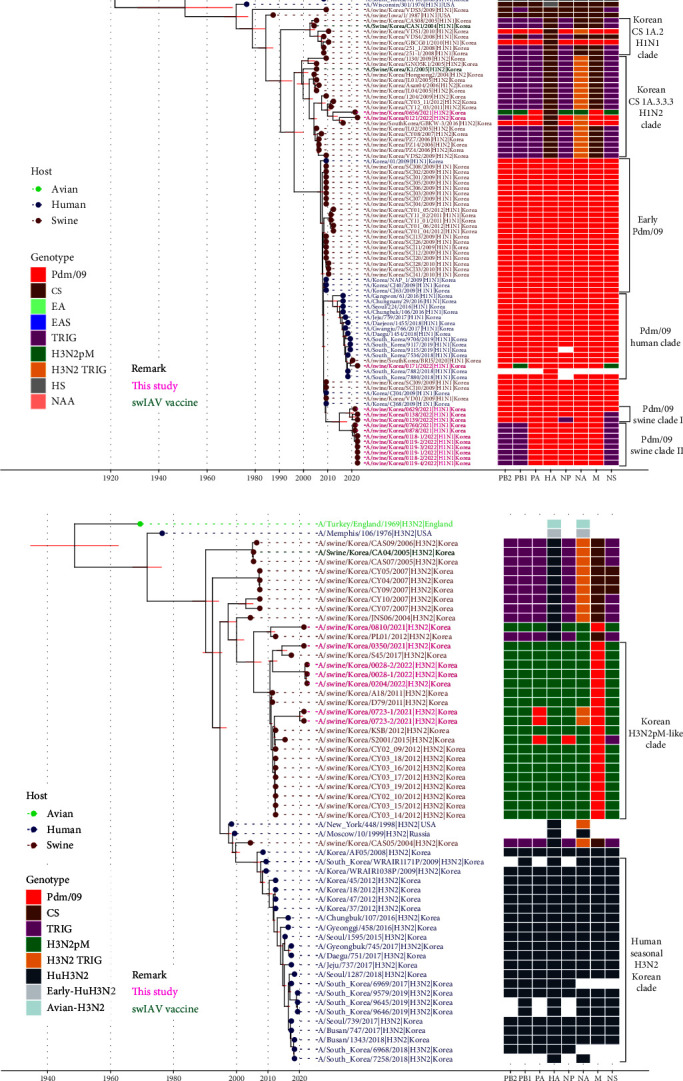
H1 and H3 swine influenza genotypes in Korea, 2004–2022. (a) Phylogenetic relationships of the H1Nx HAs. (b) Phylogenetic relationship of the H3Nx HAs. The MCC trees were reconstructed using the Bayesian evolutionary interference method. The phylogenetic branch tips with isolated hosts are color-labelled with light green (avian), dark blue (human), and dark red (swine). The branch tip labels with remarks are colored in pink (isolated in this study) and green (IAV-S vaccine used in Korea). The nodes correspond to the mean tMRCA, and the 95% HPD interval is represented with red boxes. Colored boxes show the lineage classification of each gene segment.

Among 76 Korean H1Nx IAV-S and 31 Korean H3Nx IAV-S sequences from 2004 to 2022, HA sequence data revealed that three distinct H1 lineages and two distinct H3 lineages have been circulating in Korea (Figure 1). The H1 subtype viruses included human-derived pdm09 H1N1 (37/76, 48.7%), classical swine (CS)-derived H1-TR (26/76, 34.2%), and Eurasian-avian-like swine (EAS)-H1N2 (13/76, 17.1%). Among the 23 H1Nx IAV-S isolated in this study, from 2021 to 2022, pdm09 H1N1 (12/23, 52.2%) was predominant, followed by EAS-H1N2 (9/23, 39.1%) and CS-H1N2 (2/23, 8.7%) (Figures 1(a) and 2(a)). Of note, all H1N1 IAV-S strains isolated in this study were of the pdm09 lineage, which could be divided into three distinct clades (Figures 2(a) and S2). In the pdm09 human clade, in which HA genes of human IAVs and IAV-S from the North American region have been continuously detected, two Korean IAV-S (A/swine/SouthKorea/BRI5/2020 and A/swine/Korea/0171/2022) were isolated. The pdm09 swine clade, in which the main population of Korean pdm09 H1N1 IAV-S during 2021–2022 was isolated, was newly identified in this study and could be further divided into two subclades, pdm09 swine clades I and II.

The H3-HA phylogeny revealed that H3N2 IAV-S in Korea belongs to either the H3-TR lineage (12/31, 38.7%) or the H3N2pM-like lineage (19/31, 62.3%) identified in 2011 [25]. Among the seven H3N2 IAV-S isolated in this study, the H3N2pM-like lineage (6/7, 85.7%) was dominant compared to the H3-TR lineage (1/7, 14.3%) (Figures 1(b) and 2(b)).

**Figure 3:**
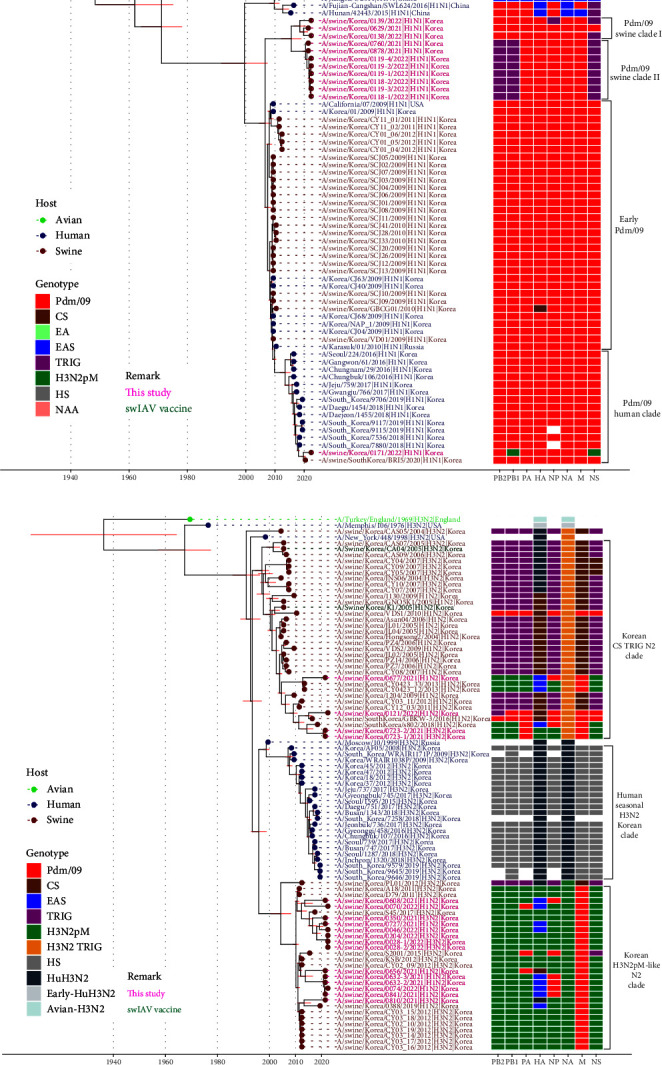
N1 and N2 swine influenza genotypes in Korea, 2004–2022. (a) Phylogenetic relationships of the HxN1 NAs. (b) Phylogenetic relationship of the HxN2 NAs. The MCC trees were reconstructed using the Bayesian evolutionary interference method. Branch tips, tip labels, and colored boxes are annotated in the same way as in Figure 2.

The phylogeny of N1-NA showed that there are two distinct N1 lineages that have been circulating in Korea, CS-H1N1 (5/43, 11.6%) and pdm09-H1N1 (38/43, 88.4%) (Figures 1(c) and 3(a)). Notably, all Korean H1N1 IAV-S detected after the introduction of pdm09 harbored the pdm09-N1 segment, while pre-2009 Korean H1N1 IAV-S contained CS-N1. The N1 gene from the H1N1 sequences isolated in this study was found in the pdm09 human clade, swine clade I and swine clade II, similar to the HA-H1 phylogeny (Figures 2(a) and 3(a)). In the N2 gene, N2 segments derived from the N2-TR lineage (35/64, 54.7%) and H3N2pM-like lineage (29/64, 45.3%) were detected. During 2021–2022, the H3N2pM-like lineage (14/18, 77.8%) was dominant compared to the TR lineage (4/14, 22.2%) (Figures 1(d) and 3(b)).

The tMRCAs for the Korean HA (H1 and H3) and NA (N1 and N2) clades were inferred by the Bayesian method using MCMC (Table 2). The tMRCA of Korean CS-H1 (H1N1 and H1N2), CS-N1 (H1N1), and TRIG-N2 (H1N2 and H3N2) were estimated to be in the 1990s, which was consistent with the previously reported prevalence of endemic TRIG H1N1, H1N2, and H3N2 in the Korean pig population before the 2009 pandemic [19, 20, 21, 22]. The tMRCAs of Korean H3N2pM-like H3 and N2 were estimated to be 2010, consistent with the emergence of H3N2pM viruses in North American and Korean regions after the 2009 pandemic [10, 25]. The tMRCA of the EAS-H1N2 H1 gene was estimated in 2012, the year before its first detection in Korea in 2013 [11, 27]. Unlike pdm09 human clade (tMRCA estimated to be 2012), the tMRCAs of pdm09 swine clade H1 and N1 were estimated as 2015, suggesting the independent evolution of pdm09 viruses within the Korean swine population [48, 49].

### 3.4. Genotypes and Antigenic Shift of IAV-S in Korea after Introduction of pdm09 Viruses

**Figure 4:**
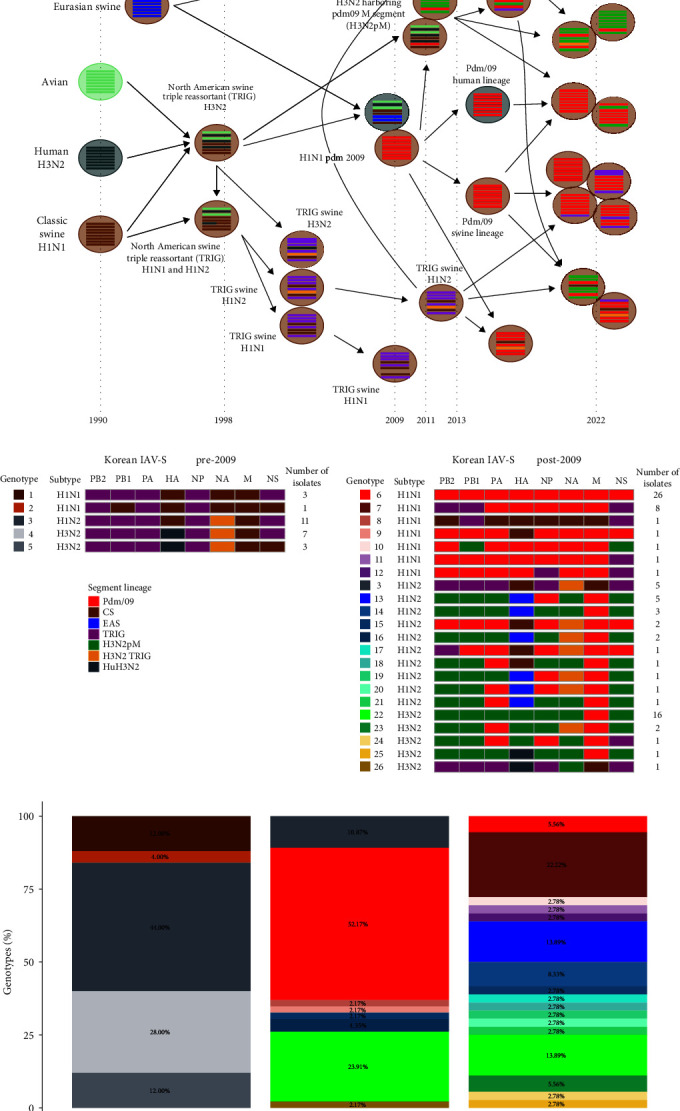
(a) Schematic representation of the genetic reassortment events that led to the development of the global A(H1N1)pdm09 pandemic and independent evolution of IAV-S in Korea. (b) Genotypes of IAV-S identified in Korea before (left) and after (right) introduction of Pdm09 into the swine population in 2009. (c) Proportional population shift of IAV-S genotypes in Korea within the periods 2004–2008 (before the 2009 pandemic), 2009–2014 (early post-2009 pandemic), and 2015–2022 (late post-2009 pandemic).

Phylogenetic analysis of each gene segment showed that the IAV-S gene pool in Korea includes genes from the pdm09 H1N1 virus, H3N2pM-like virus, EAS H1N2 virus, and TR viruses of the H1 and H3 subtypes (Figures 1, 2, 3, and 4). The overall segment reassortment events identified within the swine population of Korea from 2004 to 2022 are summarized in Figure 4(a). Of the 107 combined Korean IAV-S, 26 H1N1 IAV-S were derived entirely from the pdm09 H1N1 virus (Genotype 6), and 16 H3N2 IAV-S were derived from the H3N2pM-like virus (Genotype 22). Seventeen H1N1 (Genotypes 1–2 and 7–12), 33 H1N2 (Genotypes 3 and 13–21), and 15 H3N2 (Genotypes 4–5 and 23–26) viruses were reassortments (Figure 4(b)). Among the 65 reassortments H1N1, H1N2, and H3N2 IAV-S, we identified 26 genetic constellations, termed genotypes, 15 of which were previously reported [11, 19, 20, 22, 23, 24, 25].

The introduction of the pdm09 virus into the Korean swine population in 2009 caused a significant shift in the genetic constellation among H1N1, H1N2, and H3N2 and led to increased genotypic diversity (22 genotypes) compared to pre-2009 IAV-S (five genotypes) (Figures 4(a) and 4(b)). Of the five genotypes from the pre-2009 period, only the Genotype 3 H1N2 virus was detected post-2009. Most of the IAV-S detected in Korea since 2009 belong to Genotype 6, in which all eight gene segments originated from the human pdm09 virus (*n* = 26). The next common genotypes were Genotype 22, which consists of H3N2pM-like viruses (*n* = 16), and Genotype 13, consisting of the EAS H1N2 virus (*n* = 5) harboring the H3N2pM segments (PB2, PB1, PA, NA, M, and NS), except for the HA and NP segments, which were reassorted from the EAS H1N2 virus isolated in 2013 and the pdm09 virus, respectively (Figure 4(b)). For post-2009 H1N1, pdm09 viruses with PB2, PB1, NP, and NS segments from TR (Genotypes 7, 11, and 12) or H3N2pM-like (Genotype 10) viruses were detected sporadically. For post-2009 H1N2 and H3N2, the internal genes of pdm09 viruses and H3N2pM-like viruses were largely interspersed (Genotypes 13–25).

As shown in Figure 4(c), the period following 2009 was subdivided into two intervals, 2009–2014 and 2015–2022, according to the number of Korean IAV-S sequences that followed the introduction of the pdm09 virus (Table S1). The number of genotypes increased during the 2015–2022 period (17 genotypes) compared to the 2009–2014 period (eight genotypes), suggesting that the introduction of pdm09 into the swine population has diversified the genotypes of IAV-S in Korea.

### 3.5. Independent Evolution and Antigenic Drift within Glycoproteins of pdm09 Viruses in Korea

**Figure 5:**
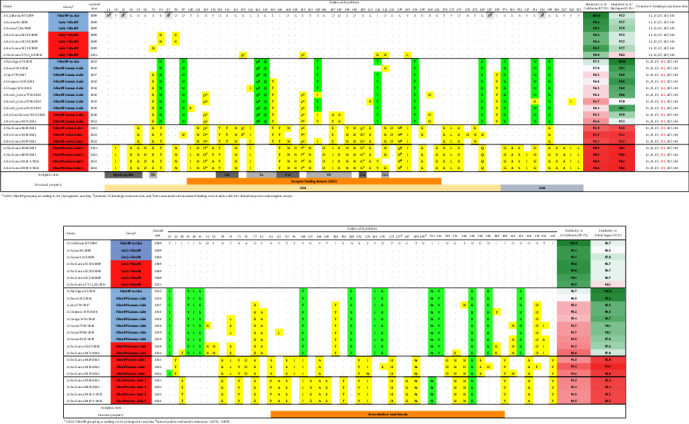
Amino acid mutations in Korean A(H1N1)pdm09 lineages. (a) Amino acid mutations in HA (H1). (b) Amino acid mutations in NA (N1). Known HA and NA antigenic sites are indicated in the tables. Dots denote positions with amino acid residues identical to the pdm09 vaccine strain A/California/07/2009. Green boxes denote positions with amino acid mutations identical to the World Health Organization (WHO)-recommended pdm09 vaccine strain A/Michigan/45/2015. Yellow boxes denote positions that are different from both vaccine strains.

As the pdm09 lineage of HA and NA segments in pdm09 was found to be separated into human and swine clades (Figures 1(a), 1(c), 2(a), and 3(a)), we analyzed the amino acid substitutions (Figure 5). HA-H1 gene sequence analysis revealed that the early pdm09 clade discovered in Korea in both human and swine populations had a high similarity to the original A/California/07/2009 H1N1 vaccine strain, with an amino acid (aa) homology of 98.0%−99.6% (Figure 5(a)). The pdm09 human clade shared similar nonsynonymous mutations with the WHO-recommended vaccine strain A/Michigan/45/2015, with aa homology ranging from 97.0% to 99.3%. Of note, two IAV-S in the pdm09 human clade, A/swine/SouthKorea/BRI5/2020 and A/swine/Korea/0171/2022, shared identical aa mutation patterns that showed high amino acid homology (96.6%−99.1%) with other pdm09 human clade IAVs isolated in the 2018–2019 season in Korea (Figure 5(a)). These data suggest that there might be an independent human-to-swine spillover of pdm09 viruses separate from the initial human-to-swine spillover in the 2009 pandemic season previously identified in Korea [23, 24]. The pdm09 human clade displayed aa homology of 94.6%−97.0% with the original A/California/07/2009 strain. Conversely, the pdm09 swine clade identified in this study, which was further divided into swine clades I and II, demonstrated independent evolution of the pdm09 virus within the Korean swine population. This was evident from the aa homology of 90.6%−92.9% and 90.2%−92.2% compared to the A/California/07/2009 strain and A/Michigan/45/2015 strain, respectively.

**Figure 6:**
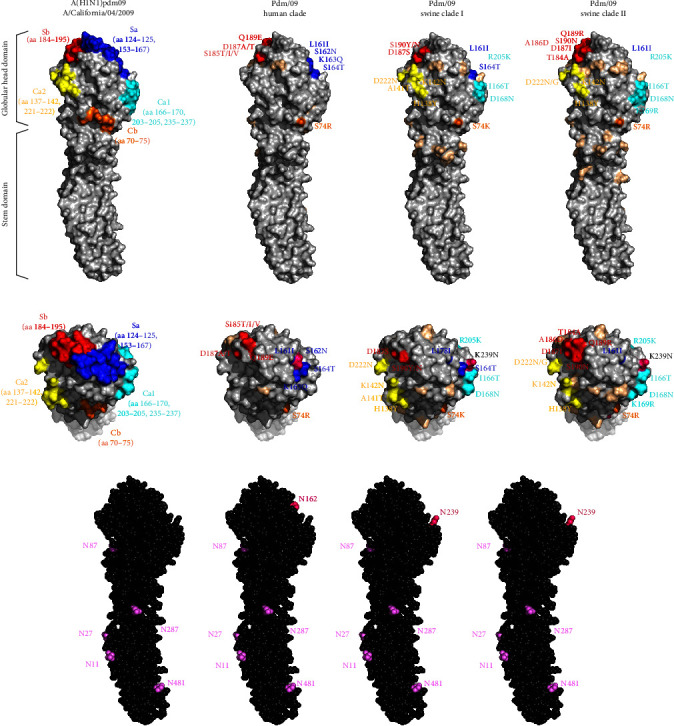
Three-dimensional structure maps of the HA monomer of the A(H1N1)pdm09 strain A/California/04/2009 (protein data bank code: 3AL4) and recent Korean pdm09 clades showing antigenic sites and amino acid substitutions as well as predicted N-linked glycosylation patterns. Five antigenic sites at the globular head of the HA1 subunit, Sa (blue), Sb (red), Ca1 (cyan), Ca2 (yellow), and Cb (orange), are marked in the structure map of A/California/04/2009 in (a) lateral view and (b) top view. Amino acid mutations at antigenic sites of the pdm09 human clade, swine clade I and swine clade II HA1 subunits are marked with the colors mentioned above, and mutations at nonantigenic sites (wheat) are also colored. (c) Predicted N-linked glycosylation sites (purple) are marked in the structure map of A/California/04/2009. Additional predicted glycosylation sites of the pdm09 human clade, swine clade I and swine clade II HA1 subunits are marked (hot pink).

Different patterns of amino acid mutations were detected in five antigenic sites (Sa, Sb, Ca1, Ca2, and Cb) in the receptor binding domain (RBD) within the globular head region of the HA1 subunit from the pdm09 human clade and swine clade (Figure 5(a)). For the human clade, amino acid mutations within the Sa site (S162N, K163Q, and S164T, H1pdm numbering) and Sb site (S185T/I/V, D187A/T, and Q189E) were detected. The IAV-S accumulated mutations within the Sa site (L161I and S164T) and Sb site (T184N, S185I, A186D, D187S/I, Q189R, and D196Y/N), but distinct mutations within the Ca1 site (I166T, D168N, K169R, and R205K) and Ca2 site (H138Y, A141T, K142N, and D222N/G) were also observed (Figures 5(a), 6(a), and 6(b)). An amino acid mutation at position 205, which was acquired by all viruses in the pdm09 swine clade, has been associated with antigenic escape [50]. Additionally, two viruses (A/swine/Korea/0629/2021 and A/swine/Korea/0138/2022) in the pdm09 swine clade acquired the HA1 mutation G155E, which has been associated with binding to *α*2,6-linked glycans in combination with the N129D mutation (a mutation acquired by all viruses of the pdm09 swine clade) and has been associated with antigenic escape [51, 52].

Independent amino acid mutations within the human clade and swine clade resulted in the acquisition of new potential N-glycosylation sites in the HA head region. The N-glycosylation sequon (N-X-T/S) is conserved at six sites in almost all H1 viruses, including pdm09 viruses. These sites include residues 11, 23, 287, and 481 of the stem region and residue 87 at the side of the globular head (Figure 6(c)). Substitutions at the Sa site (S162N and K163Q) of the human clade resulted in a potential N-glycosylation site (N162) on the top of the HA globular head, as previously described [53]. Substitution at the nonantigenic site (K239N) near the Sa and Ca1 regions of pdm09 swine clades I and II led to a new independent potential N-glycosylation site (N239) on the top of the HA globular head.

### 3.6. Sequence-Based Antigenic Cartography of Korean and Global H1 Viruses

**Figure 7:**
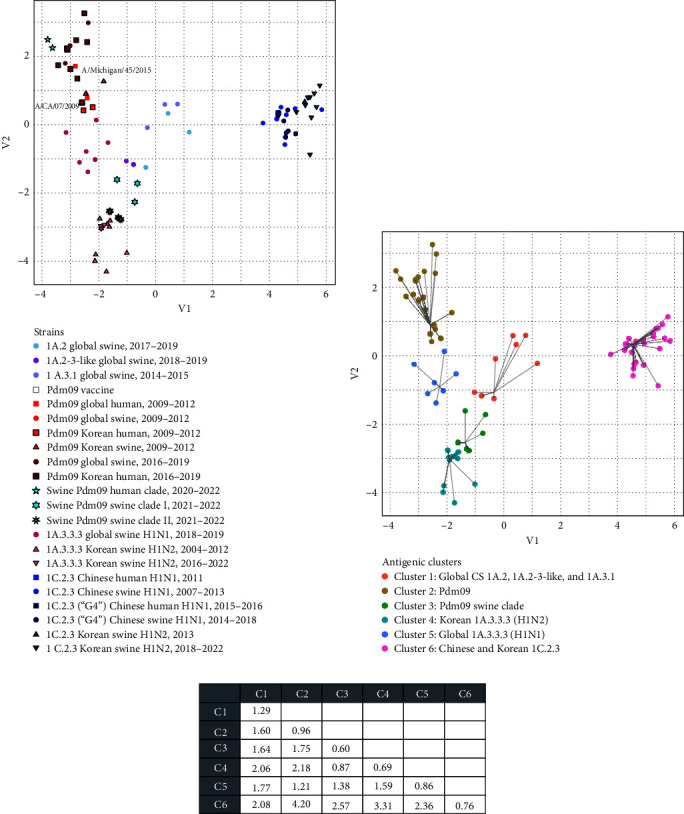
Sequence-based antigenic cartography of Korean and global swine or human H1 influenza A virus. (a) Viruses analyzed are represented as points colored according to their lineage or subclade. (b) Strains are represented as points colored and interconnected according to the assigned antigenic cluster. (c) Matrix of the mean between- and intercluster antigenic distances.

The sequence-based antigenic map of Korean and global H1 viruses exhibited six predicted clusters, which were named according to their phylogenetic, geographical, and temporal origin, through hierarchical analysis (Figure 7). Among the sequences, the mean antigenic distance was 4.3 AU, and the lowest was 0.0 AU, which was observed between viruses with a similar geographic and/or temporal origin. The highest (9.9 AU) was found between Korean swine pdm09 viruses in the pdm09 human clade (A/swine/SouthKorea/BRI5/2020 and A/swine/Korea/0171/2022) and the Chinese Eurasian-avian-like swine lineage (1C.2.3) virus (A/swine/Guangdong/1605/2010).

Among the six clusters, Korean and global pdm09 viruses antigenically close to the vaccine strains A/California/07/2009 or A/Michigan/45/2015 formed one large cluster (Cluster 2), including two viruses (A/swine/SouthKorea/BRI5/2020 and A/swine/Korea/0171/2022) identified as the result of human-to-swine spillover. However, the pdm09 swine clade identified in this study formed an independent cluster (Cluster 3) that exhibited the lowest mean distance of 0.9 AU with the recently prevailing Korean H1N2 IAV-S of classical swine lineage 1A.3.3.3 (Cluster 4). Additionally, it was antigenically closer to other clusters of classical swine lineages (Cluster 1, 1.6 AU; Cluster 5, 1.4 AU) than the pdm09 cluster (Cluster 1, 1.8 AU). Korean EAS-H1N2 viruses of the Eurasian-avian-like swine lineage (1C.2.3) prevailing since 2018 were antigenically close to Chinese 1C.2.3 H1N1 viruses or Genotype 4 (G4) viruses and formed one large cluster (Cluster 6)

### 3.7. Antigenic Cross-Reactivity with Human Influenza Vaccine Strains

Based on the HA phylogenic topology (Figure 2(a)) and sequence-based antigenic cartography (Figure 7), seven representative H1Nx viruses (two human pdm09 viruses, three swine pdm09 viruses, and two swine H1N2 viruses) were selected for antigenicity test with ferret antisera against human pdm09 vaccines A/California/07/09 and A/Michigan/45/2015 (replaced by A/Busan/383/2017). In HI assay, it was confirmed that the human-to-swine spillover H1N1pdm09 virus (A/swine/Korea/0171/2022) and the pdm09 swine clades (A/swine/Korea/0139/2022 and A/swine/Korea/0878/2021) exhibited lower cross-reactivity to A/California/07/09 and A/Busan/383/2017 (representing A/Michigan/45/2015) compared to the human H1N1pdm09 viruses A/Korea/01/2009 and A/Hawaii/70/2019 (Table 3). Consistent with the previous study [54], the Korean swine 1A.3.3.3 H1N2 virus (A/swine/Korea/0608/2021) showed limited cross-reactivity in the HI assays against ferret antiserum raised to the human seasonal vaccine strains from H1N1pdm09 (1A.3.3.2) clade. The Korean EAS 1C.2.3 H1N2 virus (A/swine/Korea/0608/2021) also showed limited cross-reactivity against the antiserum.

## 4. Discussion

Our study, conducted from 2021 to 2022, provides novel insights into the evolution and epidemiology of IAV-S in Korea, a major pork-producing country in Asia. While epidemiological information on IAV-S in Korea has been largely unknown since 2015 (Figure S1), active surveillance of IAV-S influenza by sampling all growth stages from selected farms in all provinces revealed cocirculation of H1N1, H1N2, and H3N2 IAV-S in the pig population. These findings coincide with the IAV-S subtypes that are currently circulating worldwide [40, 55, 56, 57, 58].

The genetic diversity of IAV-S in Korea prior to 2009 can be explained by the constant existence of several swine-origin H1N2 and H3N2 viruses, primarily originating in Asian and North American countries and likely introduced through the swine trade as previously described [9, 21, 23]. Since 2009, there has been evidence of the introduction of human H1N1pdm09 viruses into the swine population through reverse zoonosis in Korea (Figures 2, 3, and 4) [23, 24]. After human-to-swine spillover, there has been a significant shift in the prevalence of swine-origin IAV-S toward pdm09 viruses. In addition, endemic H3N2 viruses were reassorted with pdm09 internal genes, particularly the matrix (M) gene, resulting in the circulation of H3N2pM-like viruses within the swine population in Korea (Figure 4(c)) [10]. Additionally, following the first detection of EAS-origin H1N2 viruses harboring H3N2pM internal genes in 2013 [11], frequent reassortment was observed in major IAV-S lineages, including EAS-H1N2 and H3N2pM-like viruses, with pdm09 viruses. The IAV-S isolated in this study possess at least one gene segment from pdm09 viruses, and these frequent reassortments led to the diversification of IAV-S genotypes (Figure 4). However, no instances of EAS-H1N1 viruses or internal genes of the potentially pandemic G4 reassortant, which is widespread among the swine population in China [18], were identified.

Reverse zoonosis of pdm09 viruses by human-to-pig spillover has been frequently reported since 2009 [40, 48, 49, 59]. A significant portion of the introduced pdm09 viruses exhibit long-term persistence, extending beyond a year, and are often reassorted with endemic IAV-S [49, 60]. The antigenicity of those viruses that persist and adapt within the swine host may drift from human seasonal vaccine strains, potentially diminishing the immunity of the human population against them [49]. In this study, additional human-to-swine spillover of human pdm09 viruses in the 2018–2019 season, separate from the first spillover previously reported in the 2009 pandemic season [23, 24], was identified in Korea, in which human pdm09 viruses that were close to the WHO-recommended vaccine strain A/Michigan/45/2015 were involved (Figures 2(a) and 3(a)). The pdm09 swine clades identified in this study, distinct from both the original pdm09 virus and the recent pdm09 human clade, presumably originated from the viruses of the first spillover and persisted in the swine population for a decade with independent evolution. The analysis of amino acid mutations of pdm09 viruses in the HA1 globular head region, including antigenic sites, revealed that more frequent and diversified mutations accumulated within the pdm09 swine clade HA glycoprotein than within the human clade or original pdm09 virus (Figures 5 and 6). Among the mutations in antigenic sites, the R205K mutation was found to be associated with antigenic escape [50]. In addition to the mutations in antigenic sites, all pdm09 viruses in the swine clade as well as swine pdm09 viruses from the human clade (human-to-swine spillover viruses; A/swine/SouthKorea/BRI5/2020 and A/swine/Korea/0171/2022) shared the N129D mutation, which is located outside the major antigenic site, Sa, and this mutation is associated with not only increased binding to several *α*2,6 SA-linked glycans compared to the parental pdm09 virus but also antigenic escape [51, 52, 61]. Two viruses from swine clade I (A/swine/Korea/0629/2021 and A/swine/Korea/0138/2022) also possessed the G155E mutation, which was more frequently detected in swine pdm09 sequences (12%) than in human sequences (0.2%) from the North American dataset [49], indicating that it plays a major role in the antigenic variation of swine H1 viruses with increased affinity for *α*2,6 SA-linked glycans [51, 52]. The accumulated number of mutations within the swine pdm09 population could reduce the efficacy of current human vaccine immunity, and swine pdm09 viruses from 2019 to 2020 in the North American region, which possessed amino acid mutations found in this study (N129D, G155E, and R205K), showed significantly reduced reactivity to vaccine strains [49]. According to the sequence-based antigenic cartography of H1 viruses (Figure 7), the pdm09 swine clades identified in this study demonstrated distinct antigenicity of their HA proteins compared to pdm09 viruses in the human population, and these clades appeared to be antigenically closer to IAV-S of classical swine lineages that are predominant in the swine population. Indeed, it was confirmed in HI assays that pdm09 swine clade viruses have lower cross-reactivity to human pdm09 vaccines, and even swine pdm09 virus from the human clade showed extremely lower cross-reactivity to the vaccines compared to human pdm09 viruses (Table 3). Therefore, it is assumed that human-to-swine spillover of pdm09 viruses could lead to the generation of new pdm09 variants with altered antigenicity through independent evolution in swine, which are predicted to differ in antigenicity from existing vaccines, posing a new potential threat to public health with the emergence of new pandemic viruses.

In addition to antigenic drift, influenza viruses undergo changes in the HA structure by the addition or removal of glycosylation. Glycans near antigenic peptide epitopes interfere with antibody recognition [62], and glycans near the proteolytic activation site of HA modulate cleavage and influence the infectivity of the virus [63]. The pdm09 viruses acquired a novel N-glycosylation site at position 162 in 2014, which became predominant globally. This N-glycosylation shields epitopes recognized by antihead neutralizing antibodies, suggesting that the acquisition of N-glycosylation may be associated with immunological pressure [64, 65]. Likewise, a novel N-glycosylation site at position 239, which is located in the HA globular head region similar to N162 and near the antigenic sites Sa and Ca1, was predicted in the pdm09 viruses in the swine clade in this study (Figure 5). The occurrence of the novel N-glycosylation site within pdm09 viruses of the Korean swine population might be due to the process of host adaptation rather than immune pressure since the current IAV-S vaccine used in Korea does not match the pdm09 viruses (Figures 2 and 3) [27]. This novel N-glycosylation in pdm09 viruses may drive antigenic escape from immunity acquired by current human pdmH1N1 vaccination when IAV-S spill over into humans. Hence, the distinct evolution of pdm09 viruses within the swine population may amplify the potential impact of reintroducing these pdm09 viruses into the human population.

## 5. Conclusions

Collectively, the spillover of pdm09 viruses from humans to swine and their sustained presence within the swine population have driven a unique evolutionary trajectory for pdm09 viruses in pigs. This has also enriched the genetic diversity of swine H1N1, H1N2, and H3N2 subtypes through reassortment in Korea. Additionally, the newly introduced H1-EAS lineage viruses pose a potential public health threat, as they have the capability to evolve into a zoonotic form through reassortment with pdm09 viruses. Therefore, continuous genomic surveillance of IAVs is imperative to monitor animal-to-human spillovers and assess their zoonotic risks.

## Figures and Tables

**Table 1:** IAV-S detection from nasal swabs and pooled oral fluid specimens collected from 40 Korean swine farms from June 2021 to May 2022.

Period	Nasal swab			Pooled oral fluid		
	IAV-S positive (sample)	IAV-S positive (farm)	Virus isolation	IAV-S positive (sample)	IAV-S positive (farm)	Virus isolation
Summer 2021 (Jun.–Aug.)	2.5% (18/720)	25% (10/40)	1.0% (7/720)	5.4% (13/240)	22.5% (9/40)	0% (0/240)
Fall 2021 (Sep.–Nov.)	3.1% (22/720)	25% (10/40)	0.7% (5/720)	10.4% (25/240)	42.5% (17/40)	0.4% (1/240)
Winter 2021 (Dec.–Feb.)	6.4% (46/720)	35.0% (14/40)	0.8% (6/720)	18.3% (44/240)	45.0% (18/40)	0% (0/240)
Spring 2022 (Mar.–May)	4.6% (33/720)	32.5% (13/40)	1.4% (10/720)	15.0% (36/240)	42.5% (17/40)	0.4% (1/240)

**Table 2:** tMRCA and mean evolutionary rates of the HA and NA gene for different clades circulating in Korea, as estimated by BEAST analysis.

Group^a^	Mean tMRCA	95% HPD	Evolutionary rate^b^
H1			
CS H1N1	2,002.7	2,000.1–2,004.4	0.0032
CS H1N2	1,999.3	1,996.5–2,002.0	0.0033
pdm09	2,007.2	2,005.6–2,008.5	0.0029
pdm09 human	2,012.2	2,010.1–2,014.3	0.0034
pdm09 swine	2,015.0	2,011.7–2,018.2	0.0035
EAS H1N2	2,012.0	2,010.1–2,013.5	0.0035
H3			
H3N2 human	2,006.6	2,005.4–2,007.7	0.0033
H3N2pM-like	2,010.7	2,009.8–2,011.4	0.0034
N1			
CS H1N1	1,992.7	1,983.3–2,001.2	0.0035
pdm09	2,006.7	2,004.3–2,008.5	0.0024
pdm09 human	2,013.6	2,011.6–2,015.3	0.0030
pdm09 swine	2,015.7	2,011.9–2,018.9	0.0026
N2			
TRIG N2	1,997.0	1,994.1–1,999.6	0.0027
H3N2 human	2,007.1	2,005.8–2,008.2	0.0025
H3N2pM-like	2,010.1	2,008.9–2,011.1	0.0028

^a^Groups or clades correspond to the clades in Figures 2 and 3; ^b^calculated as substitutions/site/year.

**Table 3:** Swine and human influenza H1Nx test antigens in hemagglutination inhibition assays with monovalent ferret antisera.

Clade	Global	Antigen	Sera strain	A/California/07/09 H1N1	A/Busan/383/2017^a^ H1N1
			Clade	pdm09 human clade (vaccine)	pdm09 human clade
pdm09 human clade	1A.3.3.2	A/Korea/01/09 H1N1	—	1,024	4,092
pdm09 human clade (vaccine)	1A.3.3.2	A/Hawaii/70/2019 H1N1	—	2,046	1,024
pdm09 human clade (spillover)	1A.3.3.2	A/swine/Korea/0171/2022 H1N1	—	128	64
pdm09 swine clade I	1A.3.3.2	A/swine/Korea/0139/2022 H1N1	—	512	512
pdm09 swine clade II	1A.3.3.2	A/swine/Korea/0878/2021 H1N1	—	64	128
Korean swine 1A.3.3.3	1A.3.3.3	A/swine/Korea/0121/2022 H1N2	—	<8	<8
Korean swine 1C.2.3	1C.2.3	A/swine/Korea/0608/2021 H1N2	—	<8	<8

^a^Represents A/Michigan/45/2015 H1N1pdm09 WHO recommended vaccine.

## Data Availability

The data that support the findings of this study are available from the corresponding author upon reasonable request.

## Supplementary Materials

Supplementary Materials Table S1: the number of swine influenza A virus (H1N1, H1N2, and H3N2) sequences by subtype and isolation year used for the phylogenetic analysis. Table S2: information of Influenza A virus sequences used in this study. Table S3: GenBank accession numbers of isolated swine Influenza A virus isolated in this study. Figure S1: the number of Korean influenza A virus of swine (IAV-S) sequences used in this study. All publiclyavailable Korean IAV-S sequences were gathered as of September 2022. Figure S2: maximum-likelihood (ML) phylogenetic trees of eight segments including HA-H1, HA-H3, NA-N1, NA-N2, and internal genes (PB2, PB1, PA, NP, M, and NS). Branches are colored by lineage origin. Branch tips are shaped with circles only for sequences identified in this study. Heatmaps beside the trees indicate the origin of the host and country for each sequence. CS, classical swine; EAS, Eurasian avian-like swine; A (H1N1)pdm09, 2009 pandemic H1N1; TRIG, triple reassortant.
