# Supplementary material for: Major Shift of Influenza A Virus of Swine (IAV-S) by Human-to-Swine Spillover of the 2009 Pandemic Virus in Korea
Source: Transbound Emerg Dis. 2024 Aug 24;2024:6366170. doi: 10.1155/2024/6366170 (PMC12016703; doi:10.1155/2024/6366170)
Supplement: Supplementary Materials — Table S1: the number of swine influenza A virus (H1N1, H1N2, and H3N2) sequences by subtype and isolation year used for the phylogenetic analysis. Table S2: information of Influenza A virus sequences used in this study. Table S3: GenBank accession numbers of isolated swine Influenza A virus isolated in this study. Figure S1: the number of Korean influenza A virus of swine (IAV-S) sequences used in this study. All publiclyavailable Korean IAV-S sequences were gathered as of September 2022. Figure S2: maximum-likelihood (ML) phylogenetic trees of eight segments including HA-H1, HA-H3, NA-N1, NA-N2, and internal genes (PB2, PB1, PA, NP, M, and NS). Branches are colored by lineage origin. Branch tips are shaped with circles only for sequences identified in this study. Heatmaps beside the trees indicate the origin of the host and country for each sequence. CS, classical swine; EAS, Eurasian avian-like swine; A (H1N1)pdm09, 2009 pandemic H1N1; TRIG, triple reassortant. [file 6366170.f1.pdf]

Supplementary Table 1. The number of Swine Influenza A virus (H1N1, H1N2 and H3N2) sequences by subtype and isolation year used for the phylogenetic analysis

**Source**	**Subtype**	**pre-2004**	**2004**	**2005**	**2006**	**2007**	**2008**	**2009**	**2010**	**2011**	**2012**	**2013**	**2014**	**2015**	**2016**	**2017**	**2018**	**2019**	**2020**	**2021**	**2022**	**Total**
Korean swine IAV	H1N1	0	1	1	0	0	2	17	4	2	3	0	0	0	0	0	0	0	1	**3**[a]	**9**[a]	**43**
	H1N2	0	1	6	3	1	0	3	1	1	1	2	0	0	1	0	1	1	0	**7**[a]	**4**[a]	**33**
	H3N2	0	2	2	1	5	0	0	0	2	10	0	0	1	0	1	0	0	0	**4**[a]	**3**[a]	**31**
Korean Human IAV	H1N1	0	0	0	0	0	1	6	0	0	0	0	0	0	4	2	5	3	0	0	0	**21**
	H1N2	0	0	0	0	0	0	0	0	0	0	0	0	0	0	0	0	0	0	0	0	**0**
	H3N2	0	0	0	0	0	1	2	0	0	4	1	0	1	2	8	5	3	0	0	0	**27**
Reference	H1N1	20	1	2	0	3	2	9	5	4	5	6	7	6	7	11	14	11	0	0	0	**113**
	H1N2	0	0	0	0	0	0	0	1	0	0	0	0	0	1	1	3	2	0	0	0	**8**
	H3N2	16	0	3	4	0	1	1	7	6	5	0	1	0	3	1	7	3	0	0	0	**58**
**Total**		**36**	**5**	**14**	**8**	**9**	**7**	**38**	**18**	**15**	**28**	**9**	**8**	**8**	**18**	**24**	**35**	**23**	**1**	**14**	**16**	**334**

[a]Sequences from this study

**Supplementary Table 2.** Information of Influenza A virus sequences used in this study					
**Strain**	**Host**	**Country**	**Date**	**Source**	**Type**
A/turkey/England/1969|H3N2	Avian	England	1969	Reference avian	H3N2
A/Wisconsin/301/1976|H1N1|USA	Human	USA	1976	Reference human	H1N1
A/Memphis/106/1976|H3N2|USA	Human	USA	1976	Reference human	H3N2
A/duck/Bavaria/2/1977|H1N1|Germany	Avian	Germany	1977	Reference avian	H1N1
A/swine/Colorado/1/1977|H3N2|USA	Swine	USA	1977	Reference pig	H3N2
A/Memphis/7/1980|H1N1|USA	Human	USA	1980	Reference human	H1N1
A/New_Zealand/7/1983|H1N1|New_Zealand	Human	New_Zealand	1983	Reference human	H1N1
A/swine/Italy/670/1987|H1N1|Italy	Swine	Italy	1987	Reference pig	H1N1
A/swine/Iowa/1/1987|H1N1|USA	Swine	USA	1987	Reference pig	H1N1
A/Swine/Indiana/1726/1988|H1N1|USA	Swine	USA	1988	Reference pig	H1N1
A/swine/Memphis/1/1990|H1N1|USA	Swine	USA	1990	Reference pig	H1N1
A/swine/Denmark/19126/1993|H1N1	Swine	Denmark	1993	Reference pig	H1N1
A/swine/Denmark/WVL9/1993|H1N1	Swine	Denmark	1993	Reference pig	H1N1
A/swine/Netherlands/477/1993|H1N1	Swine	Netherlands	1993	Reference pig	H1N1
A/swine/Germany/Vi5698/95|H1N1	Swine	Germany	1995	Reference pig	H1N1
A/swine/Netherlands/609/1996|H1N1	Swine	Netherlands	1996	Reference pig	H1N1
A/swine/England/WVL16/1998|H1N1	Swine	England	1998	Reference pig	H1N1
A/swine/Texas/4199-2/1998|H3N2|USA	Swine	USA	1998	Reference pig	H3N2
A/Swine/Nebraska/209/98|H3N2|USA	Swine	USA	1998	Reference pig	H3N2
A/swine/Guangdong/01/1998|H3N2|China	Swine	China	1998	Reference pig	H3N2
A/Switzerland/7729/98|H3N2	Human	Switzerland	1998	Reference human	H3N2
A/Nagasaki/93/98|H3N2|Japan	Human	Japan	1998	Reference human	H3N2
A/New_York/448/1998|H3N2|USA	Human	USA	1998	Reference human	H3N2
A/duck/Shimane/188/1999|H1N1|Japan	Avian	Japan	1999	Reference avian	H1N1
A/green_winged_teal/Ohio/72/1999|H1N1|USA	Avian	USA	1999	Reference avian	H1N1
A/Swine/Oklahoma/18717/99|H3N2|USA	Swine	USA	1999	Reference pig	H3N2
A/Swine/Oklahoma/18089/99|H3N2|USA	Swine	USA	1999	Reference pig	H3N2
A/Swine/Wisconsin/14094/99|H3N2|USA	Swine	USA	1999	Reference pig	H3N2
A/Swine/Minnesota/593/99|H3N2|USA	Swine	USA	1999	Reference pig	H3N2
A/New_York/333/1999|H3N2|USA	Human	USA	1999	Reference human	H3N2
A/Moscow/10/1999|H3N2|Russia	Human	Russia	1999	Reference human	H3N2
A/Hong_Kong/1182/99|H3N2|China	Human	China	1999	Reference human	H3N2
A/South_Australia/40/2000|H1N1|Australia	Human	Australia	2000	Reference human	H1N1
A/New_York/310/2001|H1N1|USA	Human	USA	2001	Reference human	H1N1
A/blue_winged_teal/TX/27/2002|H1N1|USA	Avian	USA	2002	Reference avian	H1N1
A/Swine/Spain/50047/2003|H1N1|Spain	Swine	Spain	2003	Reference pig	H1N1
A/Swine/Spain/50047/2003|H1N1	Swine	Spain	2003	Reference pig	H1N1
A/swine/England/1131/2004|H1N1	Swine	England	2004	Reference pig	H1N1
A/Swine/Korea/CAN1/2004|H1N1	Swine	Korea	2004	Korea pig	H1N1
A/swine/Korea/Hongsong2/2004|H1N2|South_Korea	Swine	Korea	2004	Korea pig	H1N2
A/swine/Korea/CAS05/2004|H3N2|South_Korea	Swine	Korea	2004	Korea pig	H3N2
A/swine/Korea/JNS06/2004|H3N2|South_Korea	Swine	Korea	2004	Korea pig	H3N2
A/pintail/Alberta/68/2005|H1N1|Canada	Avian	Canada	2005	Reference human	H1N1
A/Swine/Korea/K1/2005|H1N2	Swine	Korea	2005	Korea pig	H1N2
A/swine/Korea/GNO5K1/2005|H1N2|South_Korea	Swine	Korea	2005	Korea pig	H1N2
A/swine/Korea/JL01/2005|H1N2|South_Korea	Swine	Korea	2005	Korea pig	H1N2
A/swine/Korea/JL02/2005|H1N2|South_Korea	Swine	Korea	2005	Korea pig	H1N2
A/swine/Korea/JL04/2005|H1N2|South_Korea	Swine	Korea	2005	Korea pig	H1N2
A/swine/Korea/CAS08/2005|H1N1|South_Korea	Swine	Korea	2005	Korea pig	H1N1
A/swine/Shanghai/2/2005|H1N1|China	Swine	USA	2005	Reference pig	H1N1
A/swine/British_Columbia/28103/2005|H3N2|Canada	Swine	Canada	2005	Reference pig	H3N2
A/swine/Ontario/33853/2005|H3N2|Canada	Swine	Canada	2005	Reference pig	H3N2
A/swine/Alberta/14722/2005|H3N2|Canada	Swine	Canada	2005	Reference pig	H3N2
A/Swine/Korea/CA04/2005|H3N2	Swine	Korea	2005	Korea pig	H3N2
A/swine/Korea/CAS07/2005|H3N2|South_Korea	Swine	Korea	2005	Korea pig	H3N2
A/swine/Korea/PZ14/2006|H1N2|South_Korea	Swine	Korea	2006	Korea pig	H1N2
A/swine/Korea/PZ4/2006|H1N2|South_Korea	Swine	Korea	2006	Korea pig	H1N2
A/swine/Korea/PZ7/2006|H1N2|South_Korea	Swine	Korea	2006	Korea pig	H1N2
A/swine/Korea/Asan04/2006|H1N2|South_Korea	Swine	Korea	2006	Korea pig	H1N2
A/swine/Guangdong/7/2006|H3N2|China	Swine	China	2006	Reference pig	H3N2
A/swine/Guangdong/164/06|H3N2|China	Swine	China	2006	Reference pig	H3N2
A/swine/Guangdong/166/06|H3N2|China	Swine	China	2006	Reference pig	H3N2
A/swine/Korea/CAS09/2006|H3N2|South_Korea	Swine	Korea	2006	Korea pig	H3N2
A/swine/Guangdong/223/2006|H3N2|China	Swine	China	2006	Reference pig	H3N2
A/swine/Fujian/204/2007|H1N1|China	Swine	China	2007	Reference pig	H1N1
A/swine/Zhejiang/1/2007|H1N1|China	Swine	China	2007	Reference pig	H1N1
A/swine/Hong_Kong/72/2007|H1N1|Hong_Kong	Swine	China	2007	Reference pig	H1N1
A/swine/Korea/CY08/2007|H1N2|South_Korea	Swine	Korea	2007	Korea pig	H1N2

A/swine/Korea/CY07/2007|H3N2|South_Korea	Swine	Korea	2007	Korea pig	H3N2
A/swine/Korea/CY10/2007|H3N2|South_Korea	Swine	Korea	2007	Korea pig	H3N2
A/swine/Korea/CY05/2007|H3N2|South_Korea	Swine	Korea	2007	Korea pig	H3N2
A/swine/Korea/CY04/2007|H3N2|South_Korea	Swine	Korea	2007	Korea pig	H3N2
A/swine/Korea/CY09/2007|H3N2|South_Korea	Swine	Korea	2007	Korea pig	H3N2
A/swine/Germany/SIV04/2008|H1N1	Swine	Germany	2008	Reference pig	H1N1
A/Yokohama/78/2008|H1N1|Japan	Human	Japan	2008	Reference human	H1N1
A/South_Korea/AF10/2008|H1N1|South_Korea	Human	Korea	2008	Korea human	H1N1
A/swine/Korea/VDS4/2008|H1N1|South_Korea	Swine	Korea	2008	Korea pig	H1N1
A/swine/Korea/251-1/2008|H1N1|South_Korea	Swine	Korea	2008	Korea pig	H1N1
A/Korea/AF05/2008|H3N2|South_Korea	Human	Korea	2008	Korea human	H3N2
A/swine/Hunan/3/2008|H3N2|China	Swine	China	2008	Reference pig	H3N2
A/Sichuan/1/2009|H1N1|China	Human	China	2009	Reference human	H1N1
A/Zhejiang_Yiwu/11/2009|H1N1|China	Human	China	2009	Reference human	H1N1
A/Korea/CJ63/2009|H1N1|South_Korea	Human	Korea	2009	Korea human	H1N1
A/Korea/CJ40/2009|H1N1|South_Korea	Human	Korea	2009	Korea human	H1N1
A/Korea/CJ68/2009|H1N1|South_Korea	Human	Korea	2009	Korea human	H1N1
A/Korea/CJ04/2009|H1N1|South_Korea	Human	Korea	2009	Korea human	H1N1
A/Korea/01/2009|H1N1|South_Korea	Human	Korea	2009	Korea human	H1N1
A/Korea/NAP_1/2009|H1N1|South_Korea	Human	Korea	2009	Korea human	H1N1
A/swine/Korea/1204/2009|H1N2|South_Korea	Swine	Korea	2009	Korea pig	H1N2
A/swine/Korea/1130/2009|H1N2|South_Korea	Swine	Korea	2009	Korea pig	H1N2
A/swine/Korea/VDS2/2009|H1N2|South_Korea	Swine	Korea	2009	Korea pig	H1N2
A/swine/Korea/VDS3/2009|H1N1|South_Korea	Swine	Korea	2009	Korea pig	H1N1
A/swine/Korea/SCJ01/2009|H1N1|South_Korea	Swine	Korea	2009	Korea pig	H1N1
A/swine/Korea/SCJ02/2009|H1N1|South_Korea	Swine	Korea	2009	Korea pig	H1N1
A/swine/Korea/SCJ03/2009|H1N1|South_Korea	Swine	Korea	2009	Korea pig	H1N1
A/swine/Korea/SCJ04/2009|H1N1|South_Korea	Swine	Korea	2009	Korea pig	H1N1
A/swine/Korea/SCJ05/2009|H1N1|South_Korea	Swine	Korea	2009	Korea pig	H1N1
A/swine/Korea/SCJ06/2009|H1N1|South_Korea	Swine	Korea	2009	Korea pig	H1N1
A/swine/Korea/SCJ07/2009|H1N1|South_Korea	Swine	Korea	2009	Korea pig	H1N1
A/swine/Korea/SCJ08/2009|H1N1|South_Korea	Swine	Korea	2009	Korea pig	H1N1
A/swine/Korea/SCJ09/2009|H1N1|South_Korea	Swine	Korea	2009	Korea pig	H1N1
A/swine/Korea/SCJ10/2009|H1N1|South_Korea	Swine	Korea	2009	Korea pig	H1N1
A/swine/Korea/SCJ11/2009|H1N1|South_Korea	Swine	Korea	2009	Korea pig	H1N1
A/swine/Korea/SCJ12/2009|H1N1|South_Korea	Swine	Korea	2009	Korea pig	H1N1
A/swine/Korea/SCJ13/2009|H1N1|South_Korea	Swine	Korea	2009	Korea pig	H1N1
A/swine/Korea/SCJ20/2009|H1N1|South_Korea	Swine	Korea	2009	Korea pig	H1N1
A/swine/Korea/SCJ26/2009|H1N1|South_Korea	Swine	Korea	2009	Korea pig	H1N1
A/swine/Korea/VD01/2009|H1N1|South_Korea	Swine	Korea	2009	Korea pig	H1N1
A/swine/Luxembourg/1065/2009|H1N1	Swine	Luxembourg	2009	Reference pig	H1N1
A/Mexico/4486/2009|H1N1|Mexico	Human	Mexico	2009	Reference human	H1N1
A/Taiwan/143/2009|H1N1|Taiwan	Human	Taiwan	2009	Reference human	H1N1
A/California/07/2009|H1N1|USA	Human	USA	2009	Reference human	H1N1
A/New_York/18/2009|H1N1|USA	Human	USA	2009	Reference human	H1N1
A/Wisconsin/629_D00160/2009|H1N1|USA	Human	USA	2009	Reference human	H1N1
A/Minnesota/10/2009|H1N1|USA	Human	USA	2009	Reference human	H1N1
A/Korea/WRAIR1038P/2009|H3N2|South_Korea	Human	Korea	2009	Korea human	H3N2
A/South_Korea/WRAIR1171P/2009|H3N2|South_Korea	Human	Korea	2009	Korea human	H3N2
A/chicken/Guangxi/015C10/2009|H3N2|China	Avian	China	2009	Reference avian	H3N2
A/swine/Guangdong/1623/2010|H1N1|China	Swine	China	2010	Reference pig	H1N1
A/swine/Guangdong/1605/2010|H1N1|China	Swine	China	2010	Reference pig	H1N1
A/swine/Guangdong/1/2010|H1N2|China	Swine	China	2010	Reference pig	H1N2
A/swine/Guangdong/1/2010|H1N1|China	Swine	China	2010	Reference pig	H1N1
A/swine/Gent/28/2010|H1N1|Germany	Swine	Germany	2010	Reference pig	H1N1
A/swine/Korea/GBCG01/2010|H1N1|South_Korea	Swine	Korea	2010	Korea pig	H1N1
A/swine/Korea/VDS1/2010|H1N2|South_Korea	Swine	Korea	2010	Korea pig	H1N2
A/swine/Korea/SCJ28/2010|H1N1|South_Korea	Swine	Korea	2010	Korea pig	H1N1
A/swine/Korea/SCJ33/2010|H1N1|South_Korea	Swine	Korea	2010	Korea pig	H1N1
A/swine/Korea/SCJ41/2010|H1N1|South_Korea	Swine	Korea	2010	Korea pig	H1N1
A/Karasuk/01/2010|H1N1|Russia	Human	Russia	2010	Reference human	H1N1
A/Beijing_Chaoyang/181/2010|H3N2|China	Human	China	2010	Reference human	H3N2
A/Beijing_Chaoyang/152/2010|H3N2|China	Human	China	2010	Reference human	H3N2
A/Shanghai_Baoshan/1607/2010|H3N2|China	Human	China	2010	Reference human	H3N2
A/Shantou/546/2010|H3N2|China	Human	China	2010	Reference human	H3N2
A/swine/Guangdong/L22/2010|H3N2|China	Swine	China	2010	Reference pig	H3N2
A/swine/Guangdong/L5/2010|H3N2|China	Swine	China	2010	Reference pig	H3N2
A/swine/Guangdong/L23/2010|H3N2|China	Swine	China	2010	Reference pig	H3N2
A/Jiangsu/ALS1/2011|H1N1|China	Human	China	2011	Reference human	H1N1
A/Jiangsu/1/2011|H1N1|China	Human	China	2011	Reference human	H1N1

A/swine/Jiangsu/40/2011|H1N1|China	Swine	China	2011	Reference pig	H1N1
A/swine/Italy/131916/2011|H1N1	Swine	Italy	2011	Reference pig	H1N1
A/swine/Korea/CY11_02/2011|H1N1|South_Korea	Swine	Korea	2011	Korea pig	H1N1
A/swine/Korea/CY11_01/2011|H1N1|South_Korea	Swine	Korea	2011	Korea pig	H1N1
A/swine/Korea/CY12_03/2011|H1N2|South_Korea	Swine	Korea	2011	Korea pig	H1N2
A/Victoria/361/2011|H3N2|Austrailia	Human	Australia	2011	Reference human	H3N2
A/swine/Guangxi/2242/2011|H3N2|China	Swine	China	2011	Reference pig	H3N2
A/swine/Guangxi/NS3108/2011|H3N2|China	Swine	China	2011	Reference pig	H3N2
A/swine/Guangxi/2803/2011|H3N2|China	Swine	China	2011	Reference pig	H3N2
A/swine/Korea/A18/2011|H3N2|South_Korea	Swine	Korea	2011	Korea pig	H3N2
A/swine/Korea/D79/2011|H3N2|South_Korea	Swine	Korea	2011	Korea pig	H3N2
A/swine/Guangdong/L21/2011|H3N2|China	Swine	China	2011	Reference pig	H3N2
A/duck/Guangdong/W12/2011|H3N2|China	Avian	China	2011	Reference avian	H3N2
A/swine/Shaanxi/s2/2012|H1N1|China	Swine	China	2012	Reference pig	H1N1
A/mallard/Republic_of_Georgia/1/2012|H1N1|Georgia	Avian	Georgia	2012	Reference avian	H1N1
A/swine/Germany/Ellerbrock-IDT14696/2012|H1N1	Swine	Germany	2012	Reference pig	H1N1
A/swine/Korea/CY01_06/2012|H1N1|South_Korea	Swine	Korea	2012	Korea pig	H1N1
A/swine/Korea/CY01_05/2012|H1N1|South_Korea	Swine	Korea	2012	Korea pig	H1N1
A/swine/Korea/CY03_11/2012|H1N2|South_Korea	Swine	Korea	2012	Korea pig	H1N2
A/swine/Korea/CY01_04/2012|H1N1|South_Korea	Swine	Korea	2012	Korea pig	H1N1
A/swine/Mexico/8935602/2012|H1N1	Swine	Mexico	2012	Reference pig	H1N1
A/swine/Netherlands/Dalfsen-12/2012|H1N1	Swine	Netherlands	2012	Reference pig	H1N1
A/Korea/47/2012|H3N2|South_Korea	Human	Korea	2012	Korea human	H3N2
A/Korea/18/2012|H3N2|South_Korea	Human	Korea	2012	Korea human	H3N2
A/Korea/37/2012|H3N2|South_Korea	Human	Korea	2012	Korea human	H3N2
A/Korea/45/2012|H3N2|South_Korea	Human	Korea	2012	Korea human	H3N2
A/swine/Guangxi/508/2012|H3N2|China	Swine	China	2012	Reference pig	H3N2
A/swine/Guangdong/NS2701/2012|H3N2|China	Swine	China	2012	Reference pig	H3N2
A/swine/Guangxi/NS2394/2012|H3N2|China	Swine	China	2012	Reference pig	H3N2
A/swine/Hong_Kong/2454/2012|H3N2|China	Swine	China	2012	Reference pig	H3N2
A/swine/Korea/CY03_17/2012|H3N2|South_Korea	Swine	Korea	2012	Korea pig	H3N2
A/swine/Korea/CY03_15/2012|H3N2|South_Korea	Swine	Korea	2012	Korea pig	H3N2
A/swine/Korea/CY02_10/2012|H3N2|South_Korea	Swine	Korea	2012	Korea pig	H3N2
A/swine/Korea/CY03_16/2012|H3N2|South_Korea	Swine	Korea	2012	Korea pig	H3N2
A/swine/Korea/CY03_19/2012|H3N2|South_Korea	Swine	Korea	2012	Korea pig	H3N2
A/swine/Korea/CY03_14/2012|H3N2|South_Korea	Swine	Korea	2012	Korea pig	H3N2
A/swine/Korea/CY03_18/2012|H3N2|South_Korea	Swine	Korea	2012	Korea pig	H3N2
A/swine/Korea/KSB/2012|H3N2|South_Korea	Swine	Korea	2012	Korea pig	H3N2
A/swine/Korea/CY02_09/2012|H3N2|South_Korea	Swine	Korea	2012	Korea pig	H3N2
A/swine/Korea/PL01/2012|H3N2|South_Korea	Swine	Korea	2012	Korea pig	H3N2
A/duck/Guangxi/112D4/2012|H3N2|China	Avian	China	2012	Reference avian	H3N2
A/swine/Guangxi/G2/2013|H1N1|China	Swine	China	2013	Reference pig	H1N1
A/swine/Guangxi/S2/2013|H1N1|China	Swine	China	2013	Reference pig	H1N1
A/swine/Guangdong/109/2013|H1N1|China	Swine	China	2013	Reference pig	H1N1
A/swine/Guangxi/BB1/2013|H1N1|China	Swine	China	2013	Reference pig	H1N1
A/swine/Shandong/862/2013|H1N1|China	Swine	China	2013	Reference pig	H1N1
A/swine/Jilin/625/2013|H1N1|China	Swine	China	2013	Reference pig	H1N1
A/swine/Korea/CY0423_33/2013|H1N2|South_Korea	Swine	Korea	2013	Korea pig	H1N2
A/swine/Korea/CY0423_12/2013|H1N2|South_Korea	Swine	Korea	2013	Korea pig	H1N2
A/Switzerland/9715293/2013|H3N2	Human	Switzerland	2013	Korea human	H3N2
A/swine/Shandong/S113/2014|H1N1|China	Swine	China	2014	Reference pig	H1N1
A/swine/Zhucheng/90/2014|H1N1|China	Swine	China	2014	Reference pig	H1N1
A/swine/Shandong/S93/2014|H1N1|China	Swine	China	2014	Reference pig	H1N1
A/swine/Italy/14-30549/2014|H1N1	Swine	Italy	2014	Reference pig	H1N1
A/swine/Mexico/AVX62/2014|H1N1	Swine	Mexico	2014	Reference pig	H1N1
A/mallard/Maryland/13OS3036/2014|H1N1|USA	Avian	USA	2014	Reference avian	H1N1
A/Minnesota/33/2014|H1N1|USA	Human	USA	2014	Reference human	H1N1
A/HuNan/01/2014|H3N2|China	Human	China	2014	Reference human	H3N2
A/Hunan/42443/2015|H1N1|China	Human	China	2015	Reference human	H1N1
A/swine/Guanajuato/GtoDMZC09/2015|H1N1|Mexico	Swine	Mexico	2015	Reference pig	H1N1
A/swine/Jalisco/JalDMZC05/2015|H1N1|Mexico	Swine	Mexico	2015	Reference pig	H1N1
A/duck/Mongolia/520/2015|H1N1|Mongolia	Avian	Mongolia	2015	Reference avian	H1N1
A/Iowa/39/2015|H1N1|USA	Human	USA	2015	Reference human	H1N1
A/Minnesota/46/2015|H1N1|USA	Human	USA	2015	Reference human	H1N1
A/Seoul/1595/2015|H3N2|South_Korea	Human	Korea	2015	Korea human	H3N2
A/swine/Korea/S2001/2015|H3N2|South_Korea	Swine	Korea	2015	Korea pig	H3N2
A/swine/Argentina/CIP112-2203/2016|H1N1	Swine	Argentina	2016	Reference pig	H1N1
A/swine/Manitoba/SD0148/2016|H1N2|Canada	Swine	Canada	2016	Reference pig	H1N2
A/swine/Manitoba/D0425/2016|H1N1|Canada	Swine	Canada	2016	Reference pig	H1N1
A/Fujian-cangshan/SWL624/2016|H1N1|China	Human	China	2016	Reference human	H1N1

A/swine/Shandong/9/2016|H1N1|China	Swine	China	2016	Reference pig	H1N1
A/swine/Shandong/1203/2016|H1N1|China	Swine	China	2016	Reference pig	H1N1
A/swine/Shandong/1207/2016|H1N1|China	Swine	China	2016	Reference pig	H1N1
A/Pavia/65/2016|H1N1|Italy	Human	Italy	2016	Reference human	H1N1
A/Chungnam/29/2016|H1N1|South_Korea	Human	Korea	2016	Korea human	H1N1
A/Gangwon/61/2016|H1N1|South_Korea	Human	Korea	2016	Korea human	H1N1
A/Chungbuk/106/2016|H1N1|South_Korea	Human	Korea	2016	Korea human	H1N1
A/Seoul/224/2016|H1N1|South_Korea	Human	Korea	2016	Korea human	H1N1
A/swine/SouthKorea/GBKW-3/2016|H1N2|South_Korea	Swine	Korea	2016	Korea pig	H1N2
A/Gyeonggi/458/2016|H3N2|South_Korea	Human	Korea	2016	Korea human	H3N2
A/Chungbuk/107/2016|H3N2|South_Korea	Human	Korea	2016	Korea human	H3N2
A/Michigan/96/2016|H3N2|USA	Human	USA	2016	Reference human	H3N2
A/Ohio/28/2016|H3N2|USA	Human	USA	2016	Reference human	H3N2
A/Michigan/95/2016|H3N2|USA	Human	USA	2016	Reference human	H3N2
A/swine/Quebec/DM_36/2017|H1N1|Canada	Swine	Canada	2017	Reference pig	H1N1
A/swine/Quebec/DM_28/2017|H1N1|Canada	Swine	Canada	2017	Reference pig	H1N1
A/swine/Manitoba/D0514/2017|H1N1|Canada	Swine	Canada	2017	Reference pig	H1N1
A/swine/Manitoba/D0509/2017|H1N1|Canada	Swine	Canada	2017	Reference pig	H1N1
A/swine/Heilongjiang/0140/2017|H1N1|China	Swine	China	2017	Reference pig	H1N1
A/swine/Hebei/0116/2017|H1N1|China	Swine	China	2017	Reference pig	H1N1
A/swine/Shandong/0334/2017|H1N1|China	Swine	China	2017	Reference pig	H1N1
A/swine/Heilongjiang/0110/2017|H1N1|China	Swine	China	2017	Reference pig	H1N1
A/swine/Shandong/JM78/2017|H1N1|China	Swine	China	2017	Reference pig	H1N1
A/swine/Hebei/0221/2017|H1N1|China	Swine	China	2017	Reference pig	H1N1
A/swine/Shandong/LY142/2017|H1N1|China	Swine	China	2017	Reference pig	H1N1
A/Jeju/759/2017|H1N1|South_Korea	Human	Korea	2017	Korea human	H1N1
A/Gwangju/766/2017|H1N1|South_Korea	Human	Korea	2017	Korea human	H1N1
A/Ohio/24/2017|H1N2|USA	Human	USA	2017	Reference human	H1N2
A/South_Korea/6969/2017|H3N2|South_Korea	Human	Korea	2017	Korea human	H3N2
A/Busan/747/2017|H3N2|South_Korea	Human	Korea	2017	Korea human	H3N2
A/Seoul/739/2017|H3N2|South_Korea	Human	Korea	2017	Korea human	H3N2
A/Jeju/737/2017|H3N2|South_Korea	Human	Korea	2017	Korea human	H3N2
A/Jeonbuk/736/2017|H3N2|South_Korea	Human	Korea	2017	Korea human	H3N2
A/Daegu/751/2017|H3N2|South_Korea	Human	Korea	2017	Korea human	H3N2
A/Gyeongbuk/745/2017|H3N2|South_Korea	Human	Korea	2017	Korea human	H3N2
A/Kansas/14/2017|H3N2|USA	Human	USA	2017	Korea human	H3N2
A/swine/Ohio/A01354299/2017|H3N2|USA	Swine	USA	2017	Reference pig	H3N2
A/swine/Korea/S45/2017|H3N2|South_Korea	Swine	Korea	2017	Korea pig	H3N2
A/swine/Anhui/0202/2018|H1N1|China	Swine	China	2018	Reference pig	H1N1
A/swine/Jiangsu/J006/2018|H1N1|China	Swine	China	2018	Reference pig	H1N1
A/swine/Henan/SN13/2018|H1N1|China	Swine	China	2018	Reference pig	H1N1
A/swine/Jiangsu/J005/2018|H1N1|China	Swine	China	2018	Reference pig	H1N1
A/swine/Jiangsu/J004/2018|H1N1|China	Swine	China	2018	Reference pig	H1N1
A/swine/Anhui/0203/2018|H1N1|China	Swine	China	2018	Reference pig	H1N1
A/swine/Beijing/0301/2018|H1N1|China	Swine	China	2018	Reference pig	H1N1
A/South_Korea/7536/2018|H1N1|South_Korea	Human	Korea	2018	Korea human	H1N1
A/South_Korea/7882/2018|H1N1|South_Korea	Human	Korea	2018	Korea human	H1N1
A/South_Korea/7880/2018|H1N1|South_Korea	Human	Korea	2018	Korea human	H1N1
A/Daegu/1454/2018|H1N1|South_Korea	Human	Korea	2018	Korea human	H1N1
A/Daejeon/1455/2018|H1N1|South_Korea	Human	Korea	2018	Korea human	H1N1
A/swine/SouthKorea/s802/2018|H1N2|South_Korea	Swine	Korea	2018	Korea pig	H1N2
A/swine/Spain/45690-9/2018|H1N2	Swine	Spain	2018	Reference pig	H1N2
A/swine/Ohio/A02257615/2018|H1N2|USA	Swine	USA	2018	Reference pig	H1N2
A/swine/Illinois/A02257028/2018|H1N2|USA	Swine	USA	2018	Reference pig	H1N2
A/swine/Iowa/A02257804/2018|H1N1|USA	Swine	USA	2018	Reference pig	H1N1
A/swine/Oklahoma/A01785752/2018|H1N1|USA	Swine	USA	2018	Reference pig	H1N1
A/swine/Minnesota/A01785733/2018|H1N1|USA	Swine	USA	2018	Reference pig	H1N1
A/swine/Minnesota/A01785725/2018|H1N1|USA	Swine	USA	2018	Reference pig	H1N1
A/swine/Iowa/A02257393/2018|H1N1|USA	Swine	USA	2018	Reference pig	H1N1
A/swine/Iowa/A02268960/2018|H1N1|USA	Swine	USA	2018	Reference pig	H1N1
A/swine/Illinois/A02268802/2018|H1N1|USA	Swine	USA	2018	Reference pig	H1N1
A/Seoul/1287/2018|H3N2|South_Korea	Human	Korea	2018	Korea human	H3N2
A/South_Korea/6968/2018|H3N2|South_Korea	Human	Korea	2018	Korea human	H3N2
A/Busan/1343/2018|H3N2|South_Korea	Human	Korea	2018	Korea human	H3N2
A/South_Korea/7258/2018|H3N2|South_Korea	Human	Korea	2018	Korea human	H3N2
A/Incheon/1320/2018|H3N2|South_Korea	Human	Korea	2018	Korea human	H3N2
A/Indiana/27/2018|H3N2|USA	Human	USA	2018	Reference human	H3N2
A/swine/Indiana/A02256610/2018|H3N2|USA	Swine	USA	2018	Reference pig	H3N2
A/swine/Indiana/A02427957/2018|H3N2|USA	Swine	USA	2018	Reference pig	H3N2
A/swine/Arkansas/A01678584/2018|H3N2|USA	Swine	USA	2018	Reference pig	H3N2

A/swine/Nebraska/A02268693/2018|H3N2|USA	Swine	USA	2018	Reference pig	H3N2
A/swine/Iowa/A02268957/2018|H3N2|USA	Swine	USA	2018	Reference pig	H3N2
A/swine/Minnesota/A01785743/2018|H3N2|USA	Swine	USA	2018	Reference pig	H3N2
A/South_Korea/9706/2019|H1N1|South_Korea	Human	Korea	2019	Korea human	H1N1
A/South_Korea/9115/2019|H1N1|South_Korea	Human	Korea	2019	Korea human	H1N1
A/South_Korea/9117/2019|H1N1|South_Korea	Human	Korea	2019	Korea human	H1N1
A/swine/Korea/0388/2019|H1N2	Swine	Korea	2019	Korea pig	H1N2
A/swine/Netherlands/Gent-193/2019|H1N1	Swine	Netherlands	2019	Reference pig	H1N1
A/swine/Indiana/A02429959/2019|H1N2|USA	Swine	USA	2019	Reference pig	H1N2
A/swine/Nebraska/A01785847/2019|H1N1|USA	Swine	USA	2019	Reference pig	H1N1
A/swine/Illinois/A02431144/2019|H1N1|USA	Swine	USA	2019	Reference pig	H1N1
A/swine/Ohio/A02429952/2019|H1N1|USA	Swine	USA	2019	Reference pig	H1N1
A/swine/Oklahoma/A02245006/2019|H1N2|USA	Swine	USA	2019	Reference pig	H1N2
A/swine/Oklahoma/A02245094/2019|H1N1|USA	Swine	USA	2019	Reference pig	H1N1
A/swine/Iowa/A02245107/2019|H1N1|USA	Swine	USA	2019	Reference pig	H1N1
A/swine/Illinois/A02478555/2019|H1N1|USA	Swine	USA	2019	Reference pig	H1N1
A/swine/Nebraska/A02432389/2019|H1N1|USA	Swine	USA	2019	Reference pig	H1N1
A/swine/Minnesota/A02245013/2019|H1N1|USA	Swine	USA	2019	Reference pig	H1N1
A/swine/Iowa/A02245015/2019|H1N1|USA	Swine	USA	2019	Reference pig	H1N1
A/swine/Illinois/A02431990/2019|H1N1|USA	Swine	USA	2019	Reference pig	H1N1
A/South_Korea/9645/2019|H3N2|South_Korea	Human	Korea	2019	Korea human	H3N2
A/South_Korea/9646/2019|H3N2|South_Korea	Human	Korea	2019	Korea human	H3N2
A/South_Korea/9579/2019|H3N2|South_Korea	Human	Korea	2019	Korea human	H3N2
A/swine/Iowa/A02478539/2019|H3N2|USA	Swine	USA	2019	Reference pig	H3N2
A/swine/Minnesota/A02245182/2019|H3N2|USA	Swine	USA	2019	Reference pig	H3N2
A/swine/Iowa/A02431988/2019|H3N2|USA	Swine	USA	2019	Reference pig	H3N2
A/swine/SouthKorea/BRI5/2020|H1N1|South_Korea	Swine	Korea	2020	Korea pig	H1N1
A/swine/Korea/0608/2021|H1N2	Swine	Korea	2021	This study	H1N2
A/swine/Korea/0629/2021|H1N1	Swine	Korea	2021	This study	H1N1
A/swine/Korea/0632-3/2021|H1N2	Swine	Korea	2021	This study	H1N2
A/swine/Korea/0677/2021|H1N2	Swine	Korea	2021	This study	H1N2
A/swine/Korea/0727/2021|H1N2	Swine	Korea	2021	This study	H1N2
A/swine/Korea/0760/2021|H1N1	Swine	Korea	2021	This study	H1N1
A/swine/Korea/0632-2/2021|H1N2	Swine	Korea	2021	This study	H1N2
A/swine/Korea/0656/2021|H1N2	Swine	Korea	2021	This study	H1N2
A/swine/Korea/0841/2021|H1N2	Swine	Korea	2021	This study	H1N2
A/swine/Korea/0878/2021|H1N1	Swine	Korea	2021	This study	H1N1
A/swine/Korea/0723-1/2021|H3N2	Swine	Korea	2021	This study	H3N2
A/swine/Korea/0723-2/2021|H3N2	Swine	Korea	2021	This study	H3N2
A/swine/Korea/0350/2021|H3N2	Swine	Korea	2021	This study	H3N2
A/swine/Korea/0810/2021|H3N2	Swine	Korea	2021	This study	H3N2
A/swine/Korea/0046/2022|H1N2	Swine	Korea	2022	This study	H1N2
A/swine/Korea/0070/2022|H1N2	Swine	Korea	2022	This study	H1N2
A/swine/Korea/0074/2022|H1N2	Swine	Korea	2022	This study	H1N2
A/swine/Korea/0118-1/2022|H1N1	Swine	Korea	2022	This study	H1N1
A/swine/Korea/0118-2/2022|H1N1	Swine	Korea	2022	This study	H1N1
A/swine/Korea/0119-1/2022|H1N1	Swine	Korea	2022	This study	H1N1
A/swine/Korea/0119-2/2022|H1N1	Swine	Korea	2022	This study	H1N1
A/swine/Korea/0119-3/2022|H1N1	Swine	Korea	2022	This study	H1N1
A/swine/Korea/0119-4/2022|H1N1	Swine	Korea	2022	This study	H1N1
A/swine/Korea/0121/2022|H1N2	Swine	Korea	2022	This study	H1N2
A/swine/Korea/0138/2022|H1N1	Swine	Korea	2022	This study	H1N1
A/swine/Korea/0139/2022|H1N1	Swine	Korea	2022	This study	H1N1
A/swine/Korea/0171/2022|H1N1	Swine	Korea	2022	This study	H1N1
A/swine/Korea/0204/2022|H3N2	Swine	Korea	2022	This study	H3N2
A/swine/Korea/0028-1/2022|H3N2	Swine	Korea	2022	This study	H3N2
A/swine/Korea/0028-2/2022|H3N2	Swine	Korea	2022	This study	H3N2

**Supplementary Table 3.** GenBank accession numbers of isolated swine Influenza A virus isolated in this study									
Sequence	Subtype	PB2	PB1	PA	HA	NP	NA	M	NS
A/swine/Korea/0028-1/2022	H3N2	OR762281	OR762312	OR762343	OR733507	OR762374	OR762405	OR762436	OR762467
A/swine/Korea/0028-2/2022	H3N2	OR762282	OR762313	OR762344	OR733508	OR762375	OR762406	OR762437	OR762468
A/swine/Korea/0046/2022	H1N2	OR762283	OR762314	OR762345	OR733514	OR762376	OR762407	OR762438	OR762469
A/swine/Korea/0070/2022	H1N2	OR762284	OR762315	OR762346	OR733515	OR762377	OR762408	OR762439	OR762470
A/swine/Korea/0074/2022	H1N2	OR762285	OR762316	OR762347	OR733516	OR762378	OR762409	OR762440	OR762471
A/swine/Korea/0118-1/2022	H1N1	OR762286	OR762317	OR762348	OR733526	OR762379	OR762410	OR762441	OR762472
A/swine/Korea/0118-2/2022	H1N1	OR762287	OR762318	OR762349	OR733527	OR762380	OR762411	OR762442	OR762473
A/swine/Korea/0119-1/2022	H1N1	OR762288	OR762319	OR762350	OR733528	OR762381	OR762412	OR762443	OR762474
A/swine/Korea/0119-2/2022	H1N1	OR762289	OR762320	OR762351	OR733529	OR762382	OR762413	OR762444	OR762475
A/swine/Korea/0119-3/2022	H1N1	OR762290	OR762321	OR762352	OR733530	OR762383	OR762414	OR762445	OR762476
A/swine/Korea/0119-4/2022	H1N1	OR762291	OR762322	OR762353	OR733531	OR762384	OR762415	OR762446	OR762477
A/swine/Korea/0121/2022	H1N2	OR762292	OR762323	OR762354	OR733517	OR762385	OR762416	OR762447	OR762478
A/swine/Korea/0138/2022	H1N1	OR762293	OR762324	OR762355	OR733532	OR762386	OR762417	OR762448	OR762479
A/swine/Korea/0139/2022	H1N1	OR762294	OR762325	OR762356	OR733533	OR762387	OR762418	OR762449	OR762480
A/swine/Korea/0171/2022	H1N1	OR762295	OR762326	OR762357	OR733534	OR762388	OR762419	OR762450	OR762481
A/swine/Korea/0204/2022	H3N2	OR762296	OR762327	OR762358	OR733509	OR762389	OR762420	OR762451	OR762482
A/swine/Korea/0350/2021	H3N2	OR762267	OR762298	OR762329	OR733510	OR762360	OR762391	OR762422	OR762453
A/swine/Korea/0388/2019	H1N2	OR762266	OR762297	OR762328	OR733518	OR762359	OR762390	OR762421	OR762452
A/swine/Korea/0608/2021	H1N2	OR762268	OR762299	OR762330	OR733519	OR762361	OR762392	OR762423	OR762454
A/swine/Korea/0629/2021	H1N1	OR762269	OR762300	OR762331	OR733535	OR762362	OR762393	OR762424	OR762455
A/swine/Korea/0632-2/2021	H1N2	OR762277	OR762308	OR762339	OR733520	OR762370	OR762401	OR762432	OR762463
A/swine/Korea/0632-3/2021	H1N2	OR762270	OR762301	OR762332	OR733521	OR762363	OR762394	OR762425	OR762456
A/swine/Korea/0656/2021	H1N2	OR762278	OR762309	OR762340	OR733522	OR762371	OR762402	OR762433	OR762464
A/swine/Korea/0677/2021	H1N2	OR762271	OR762302	OR762333	OR733523	OR762364	OR762395	OR762426	OR762457
A/swine/Korea/0723-1/2021	H3N2	OR762272	OR762303	OR762334	OR733511	OR762365	OR762396	OR762427	OR762458
A/swine/Korea/0723-2/2021	H3N2	OR762273	OR762304	OR762335	OR733512	OR762366	OR762397	OR762428	OR762459
A/swine/Korea/0727/2021	H1N2	OR762274	OR762305	OR762336	OR733524	OR762367	OR762398	OR762429	OR762460
A/swine/Korea/0760/2021	H1N1	OR762275	OR762306	OR762337	OR733536	OR762368	OR762399	OR762430	OR762461
A/swine/Korea/0810/2021	H3N2	OR762276	OR762307	OR762338	OR733513	OR762369	OR762400	OR762431	OR762462
A/swine/Korea/0841/2021	H1N2	OR762279	OR762310	OR762341	OR733525	OR762372	OR762403	OR762434	OR762465
A/swine/Korea/0878/2021	H1N1	OR762280	OR762311	OR762342	OR733537	OR762373	OR762404	OR762435	OR762466

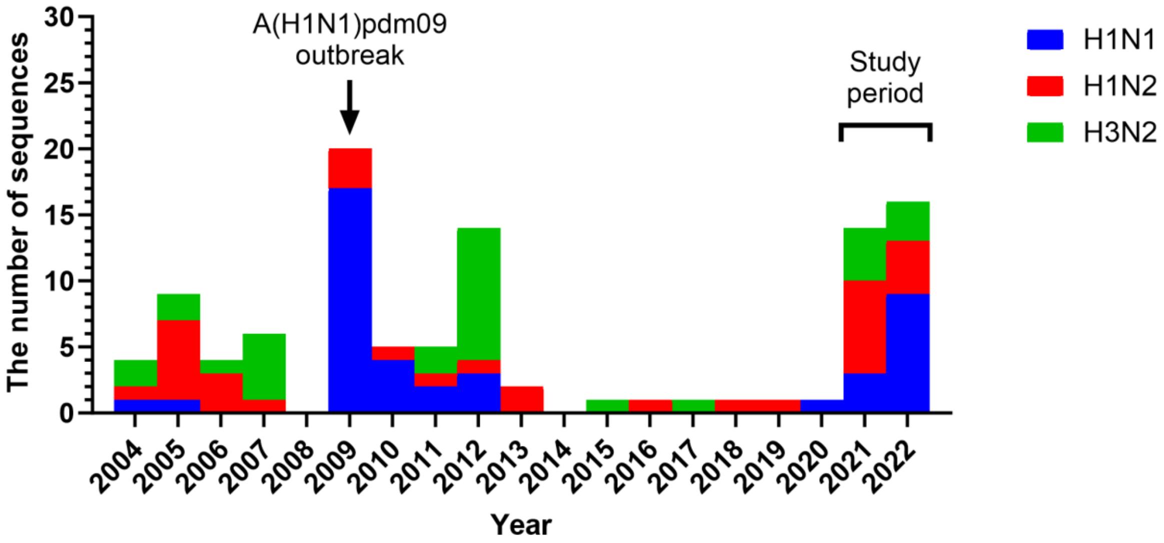

Supplementary Figure 1. The number of Korean influenza A virus of swine (IAV-S) sequences used in this study. All publicly available Korean IAV-S sequences were gathered as of September 2022.

HA (H1)

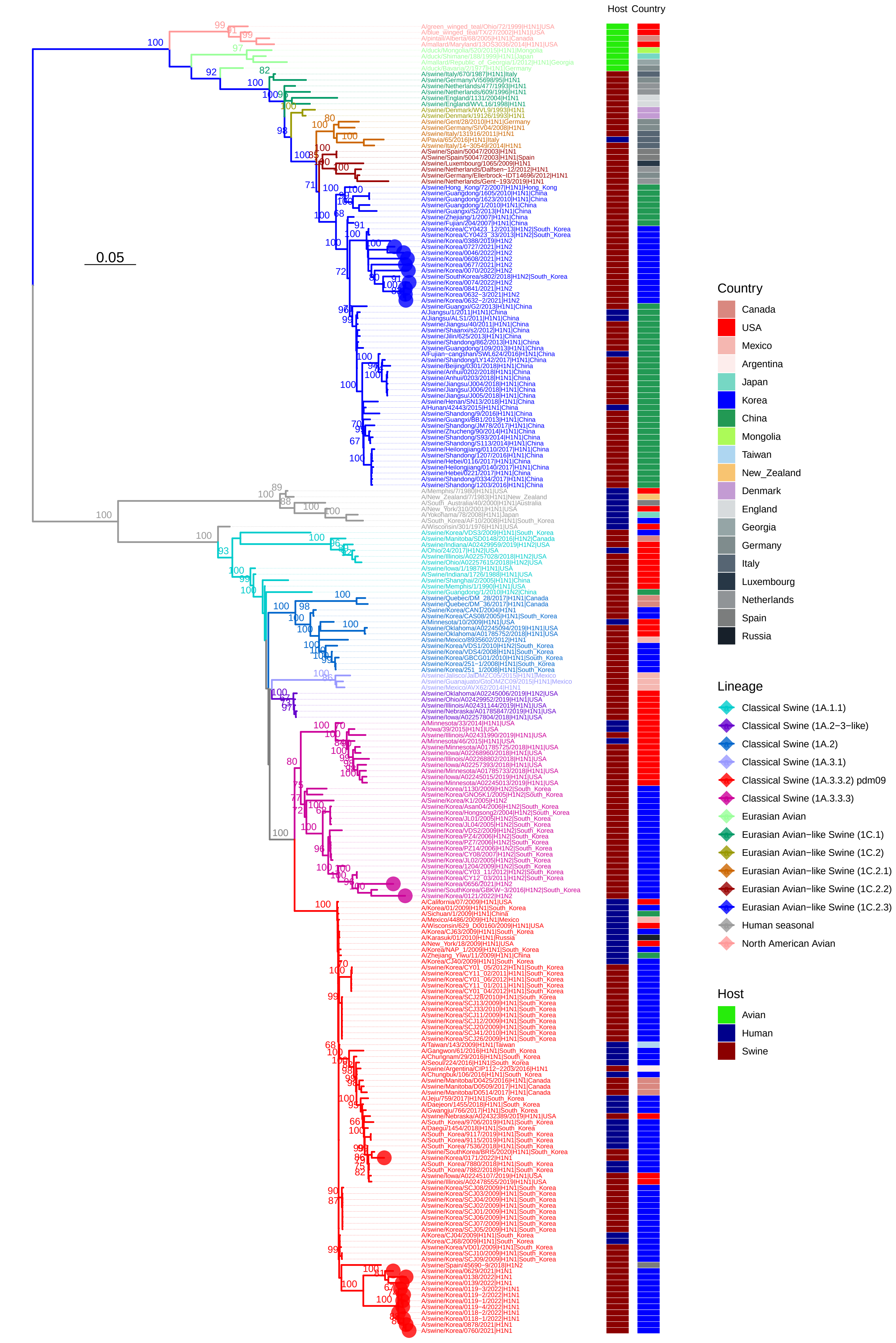

Host Country
0.05
Country
Canada
USA
Mexico
Argentina
Japan
Korea
China
Mongolia
Taiwan
New_Zealand
Denmark
England
Georgia
Germany
Italy
Luxembourg
Netherlands
Spain
Russia
Lineage
Classical Swine (1A.1.1)
Classical Swine (1A.2−3−like)
Classical Swine (1A.2)
Classical Swine (1A.3.1)
Classical Swine (1A.3.3.2) pdm09
Classical Swine (1A.3.3.3)
Eurasian Avian
Eurasian Avian−like Swine (1C.1)
Eurasian Avian−like Swine (1C.2)
Eurasian Avian−like Swine (1C.2.1)
Eurasian Avian−like Swine (1C.2.2)
Eurasian Avian−like Swine (1C.2.3)
Human seasonal
North American Avian
Host
Avian
Human
Swine

HA (H3)

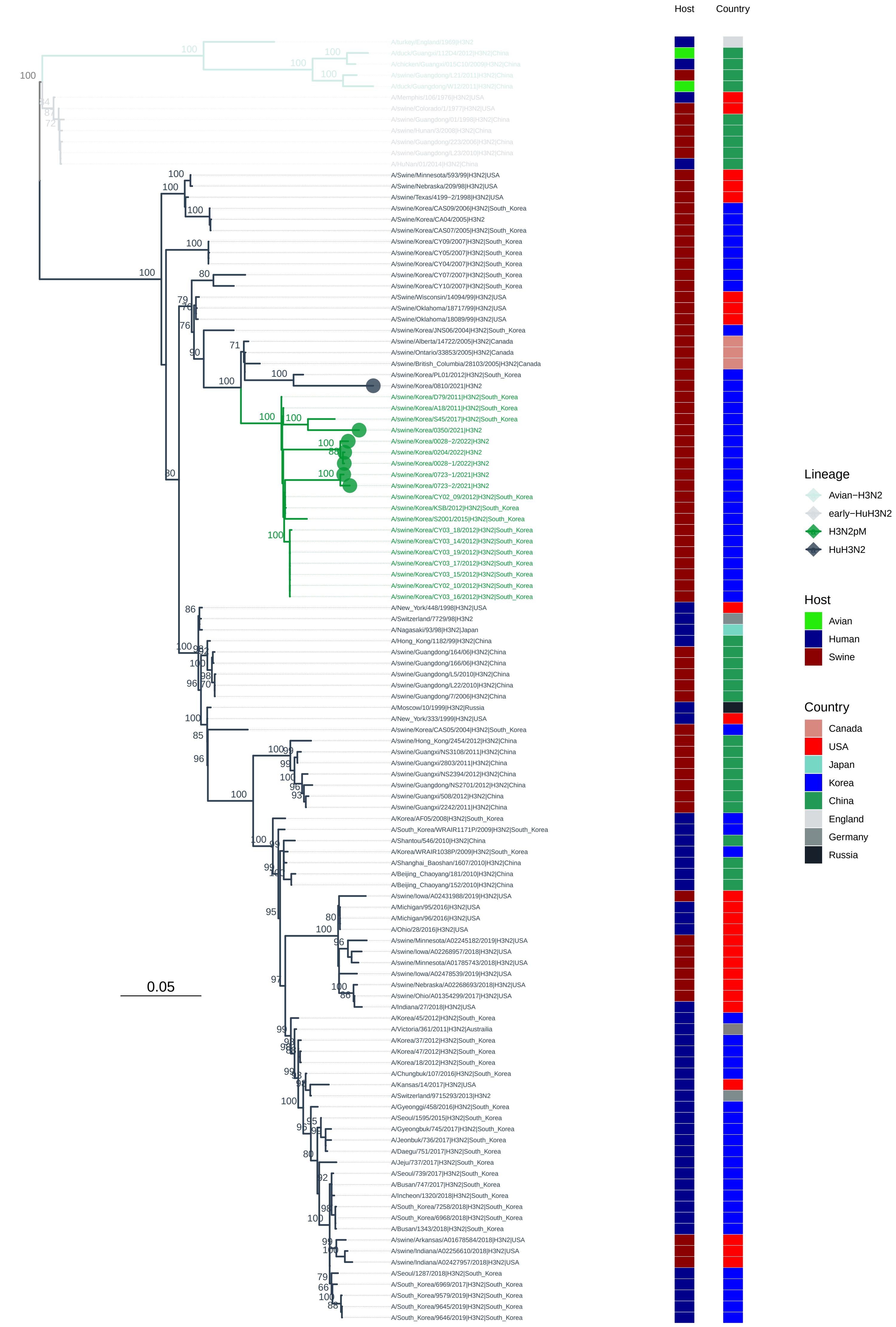

Host
Country
A/turkey/England/1969|H3N2
A/duck/Guangxi/112D4/2012|H3N2|China
A/chicken/Guangxi/015C10/2009|H3N2|China
A/swine/Guangdong/L21/2011|H3N2|China
A/duck/Guangdong/W12/2011|H3N2|China
A/Memphis/106/1976|H3N2|USA
A/swine/Colorado/1/1977|H3N2|USA
A/swine/Guangdong/01/1998|H3N2|China
A/swine/Hunan/3/2008|H3N2|China
A/swine/Guangdong/223/2006|H3N2|China
A/swine/Guangdong/L23/2010|H3N2|China
A/HuNan/01/2014|H3N2|China
A/Swine/Minnesota/593/99|H3N2|USA
A/Swine/Nebraska/209/98|H3N2|USA
A/swine/Texas/4199−2/1998|H3N2|USA
A/swine/Korea/CAS09/2006|H3N2|South_Korea
A/Swine/Korea/CA04/2005|H3N2
A/swine/Korea/CAS07/2005|H3N2|South_Korea
A/swine/Korea/CY09/2007|H3N2|South_Korea
A/swine/Korea/CY05/2007|H3N2|South_Korea
A/swine/Korea/CY04/2007|H3N2|South_Korea
A/swine/Korea/CY07/2007|H3N2|South_Korea
A/swine/Korea/CY10/2007|H3N2|South_Korea
A/Swine/Wisconsin/14094/99|H3N2|USA
A/Swine/Oklahoma/18717/99|H3N2|USA
A/Swine/Oklahoma/18089/99|H3N2|USA
A/swine/Korea/JNS06/2004|H3N2|South_Korea
A/swine/Alberta/14722/2005|H3N2|Canada
A/swine/Ontario/33853/2005|H3N2|Canada
A/swine/British_Columbia/28103/2005|H3N2|Canada
A/swine/Korea/PL01/2012|H3N2|South_Korea
A/swine/Korea/0810/2021|H3N2
A/swine/Korea/D79/2011|H3N2|South_Korea
A/swine/Korea/A18/2011|H3N2|South_Korea
A/swine/Korea/S45/2017|H3N2|South_Korea
A/swine/Korea/0350/2021|H3N2
A/swine/Korea/0028−2/2022|H3N2
A/swine/Korea/0204/2022|H3N2
A/swine/Korea/0028−1/2022|H3N2
A/swine/Korea/0723−1/2021|H3N2
A/swine/Korea/0723−2/2021|H3N2
A/swine/Korea/CY02_09/2012|H3N2|South_Korea
A/swine/Korea/KSB/2012|H3N2|South_Korea
A/swine/Korea/S2001/2015|H3N2|South_Korea
A/swine/Korea/CY03_18/2012|H3N2|South_Korea
A/swine/Korea/CY03_14/2012|H3N2|South_Korea
A/swine/Korea/CY03_19/2012|H3N2|South_Korea
A/swine/Korea/CY03_17/2012|H3N2|South_Korea
A/swine/Korea/CY03_15/2012|H3N2|South_Korea
A/swine/Korea/CY02_10/2012|H3N2|South_Korea
A/swine/Korea/CY03_16/2012|H3N2|South_Korea
A/New_York/448/1998|H3N2|USA
A/Switzerland/7729/98|H3N2
A/Nagasaki/93/98|H3N2|Japan
A/Hong_Kong/1182/99|H3N2|China
A/swine/Guangdong/164/06|H3N2|China
A/swine/Guangdong/166/06|H3N2|China
A/swine/Guangdong/L5/2010|H3N2|China
A/swine/Guangdong/L22/2010|H3N2|China
A/swine/Guangdong/7/2006|H3N2|China
A/Moscow/10/1999|H3N2|Russia
A/New_York/333/1999|H3N2|USA
A/swine/Korea/CAS05/2004|H3N2|South_Korea
A/swine/Hong_Kong/2454/2012|H3N2|China
A/swine/Guangxi/NS3108/2011|H3N2|China
A/swine/Guangxi/2803/2011|H3N2|China
A/swine/Guangxi/NS2394/2012|H3N2|China
A/swine/Guangdong/NS2701/2012|H3N2|China
A/swine/Guangxi/508/2012|H3N2|China
A/swine/Guangxi/2242/2011|H3N2|China
A/Korea/AF05/2008|H3N2|South_Korea
A/South_Korea/WRAIR1171P/2009|H3N2|South_Korea
A/Shantou/546/2010|H3N2|China
A/Korea/WRAIR1038P/2009|H3N2|South_Korea
A/Shanghai_Baoshan/1607/2010|H3N2|China
A/Beijing_Chaoyang/181/2010|H3N2|China
A/Beijing_Chaoyang/152/2010|H3N2|China
A/swine/Iowa/A02431988/2019|H3N2|USA
A/Michigan/95/2016|H3N2|USA
A/Michigan/96/2016|H3N2|USA
A/Ohio/28/2016|H3N2|USA
A/swine/Minnesota/A02245182/2019|H3N2|USA
A/swine/Iowa/A02268957/2018|H3N2|USA
A/swine/Minnesota/A01785743/2018|H3N2|USA
A/swine/Iowa/A02478539/2019|H3N2|USA
A/swine/Nebraska/A02268693/2018|H3N2|USA
A/swine/Ohio/A01354299/2017|H3N2|USA
A/Indiana/27/2018|H3N2|USA
A/Korea/45/2012|H3N2|South_Korea
A/Victoria/361/2011|H3N2|Austrailia
A/Korea/37/2012|H3N2|South_Korea
A/Korea/47/2012|H3N2|South_Korea
A/Korea/18/2012|H3N2|South_Korea
A/Chungbuk/107/2016|H3N2|South_Korea
A/Kansas/14/2017|H3N2|USA
A/Switzerland/9715293/2013|H3N2
A/Gyeonggi/458/2016|H3N2|South_Korea
A/Seoul/1595/2015|H3N2|South_Korea
A/Gyeongbuk/745/2017|H3N2|South_Korea
A/Jeonbuk/736/2017|H3N2|South_Korea
A/Daegu/751/2017|H3N2|South_Korea
A/Jeju/737/2017|H3N2|South_Korea
A/Seoul/739/2017|H3N2|South_Korea
A/Busan/747/2017|H3N2|South_Korea
A/Incheon/1320/2018|H3N2|South_Korea
A/South_Korea/7258/2018|H3N2|South_Korea
A/South_Korea/6968/2018|H3N2|South_Korea
A/Busan/1343/2018|H3N2|South_Korea
A/swine/Arkansas/A01678584/2018|H3N2|USA
A/swine/Indiana/A02256610/2018|H3N2|USA
A/swine/Indiana/A02427957/2018|H3N2|USA
A/Seoul/1287/2018|H3N2|South_Korea
A/South_Korea/6969/2017|H3N2|South_Korea
A/South_Korea/9579/2019|H3N2|South_Korea
A/South_Korea/9645/2019|H3N2|South_Korea
A/South_Korea/9646/2019|H3N2|South_Korea
0.05
Lineage
Avian−H3N2
early−HuH3N2
H3N2pM
HuH3N2
Host
Avian
Human
Swine
Country
Canada
USA
Japan
Korea
China
England
Germany
Russia

NA (N1)

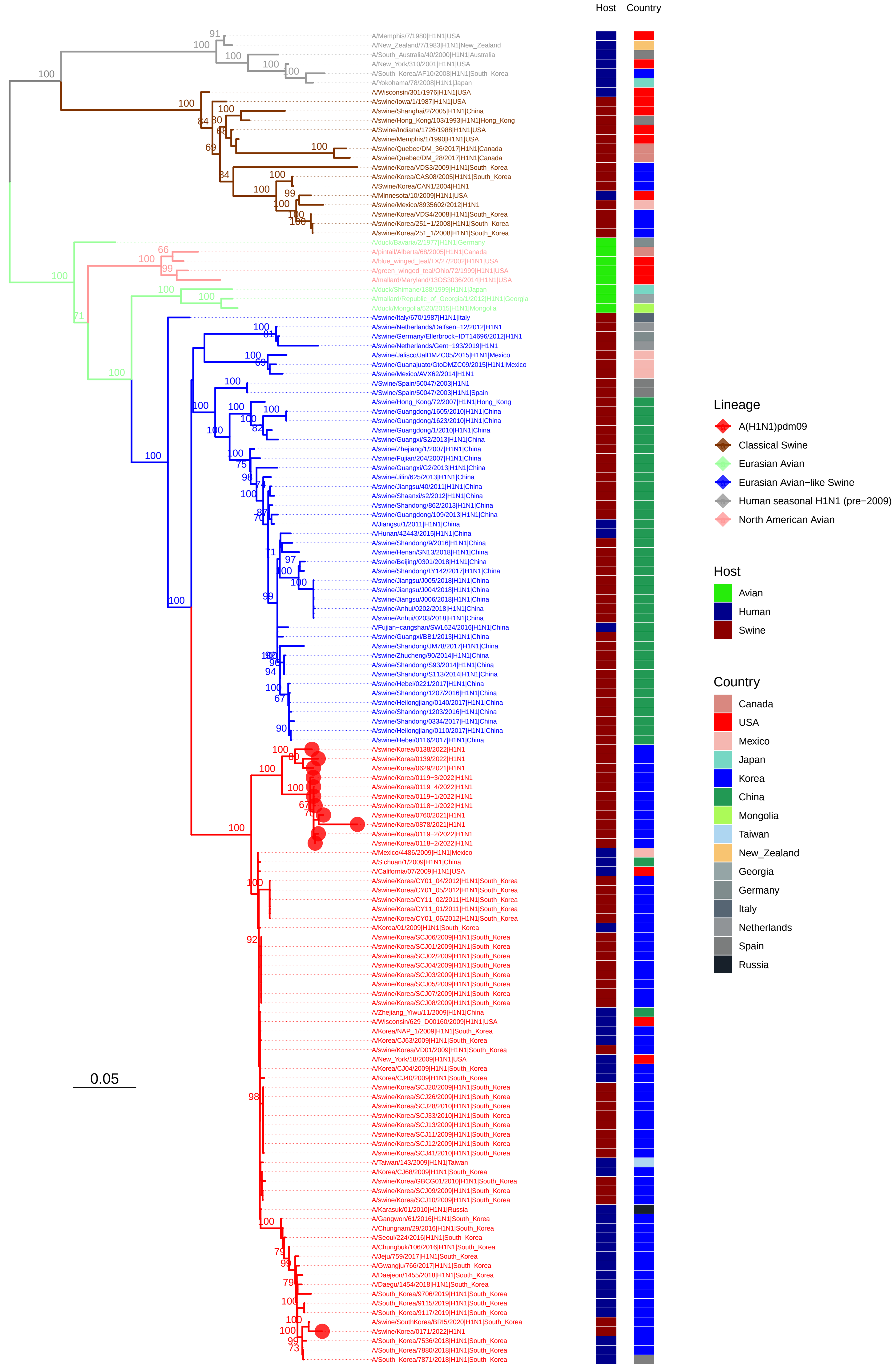

Host
Country
A/Memphis/7/1980|H1N1|USA
A/New_Zealand/7/1983|H1N1|New_Zealand
A/South_Australia/40/2000|H1N1|Australia
A/New_York/310/2001|H1N1|USA
A/South_Korea/AF10/2008|H1N1|South_Korea
A/Yokohama/78/2008|H1N1|Japan
A/Wisconsin/301/1976|H1N1|USA
A/swine/Iowa/1/1987|H1N1|USA
A/swine/Shanghai/2/2005|H1N1|China
A/swine/Hong_Kong/103/1993|H1N1|Hong_Kong
A/Swine/Indiana/1726/1988|H1N1|USA
A/swine/Memphis/1/1990|H1N1|USA
A/swine/Quebec/DM_36/2017|H1N1|Canada
A/swine/Quebec/DM_28/2017|H1N1|Canada
A/swine/Korea/VDS3/2009|H1N1|South_Korea
A/swine/Korea/CAS08/2005|H1N1|South_Korea
A/Swine/Korea/CAN1/2004|H1N1
A/Minnesota/10/2009|H1N1|USA
A/swine/Mexico/8935602/2012|H1N1
A/swine/Korea/VDS4/2008|H1N1|South_Korea
A/swine/Korea/251−1/2008|H1N1|South_Korea
A/swine/Korea/251_1/2008|H1N1|South_Korea
A/duck/Bavaria/2/1977|H1N1|Germany
A/pintail/Alberta/68/2005|H1N1|Canada
A/blue_winged_teal/TX/27/2002|H1N1|USA
A/green_winged_teal/Ohio/72/1999|H1N1|USA
A/mallard/Maryland/13OS3036/2014|H1N1|USA
A/duck/Shimane/188/1999|H1N1|Japan
A/mallard/Republic_of_Georgia/1/2012|H1N1|Georgia
A/duck/Mongolia/520/2015|H1N1|Mongolia
A/swine/Italy/670/1987|H1N1|Italy
A/swine/Netherlands/Dalfsen−12/2012|H1N1
A/swine/Germany/Ellerbrock−IDT14696/2012|H1N1
A/swine/Netherlands/Gent−193/2019|H1N1
A/swine/Jalisco/JalDMZC05/2015|H1N1|Mexico
A/swine/Guanajuato/GtoDMZC09/2015|H1N1|Mexico
A/swine/Mexico/AVX62/2014|H1N1
A/Swine/Spain/50047/2003|H1N1
A/Swine/Spain/50047/2003|H1N1|Spain
A/swine/Hong_Kong/72/2007|H1N1|Hong_Kong
A/swine/Guangdong/1605/2010|H1N1|China
A/swine/Guangdong/1623/2010|H1N1|China
A/swine/Guangdong/1/2010|H1N1|China
A/swine/Guangxi/S2/2013|H1N1|China
A/swine/Zhejiang/1/2007|H1N1|China
A/swine/Fujian/204/2007|H1N1|China
A/swine/Guangxi/G2/2013|H1N1|China
A/swine/Jilin/625/2013|H1N1|China
A/swine/Jiangsu/40/2011|H1N1|China
A/swine/Shaanxi/s2/2012|H1N1|China
A/swine/Shandong/862/2013|H1N1|China
A/swine/Guangdong/109/2013|H1N1|China
A/Jiangsu/1/2011|H1N1|China
A/Hunan/42443/2015|H1N1|China
A/swine/Shandong/9/2016|H1N1|China
A/swine/Henan/SN13/2018|H1N1|China
A/swine/Beijing/0301/2018|H1N1|China
A/swine/Shandong/LY142/2017|H1N1|China
A/swine/Jiangsu/J005/2018|H1N1|China
A/swine/Jiangsu/J004/2018|H1N1|China
A/swine/Jiangsu/J006/2018|H1N1|China
A/swine/Anhui/0202/2018|H1N1|China
A/swine/Anhui/0203/2018|H1N1|China
A/Fujian−cangshan/SWL624/2016|H1N1|China
A/swine/Guangxi/BB1/2013|H1N1|China
A/swine/Shandong/JM78/2017|H1N1|China
A/swine/Zhucheng/90/2014|H1N1|China
A/swine/Shandong/S93/2014|H1N1|China
A/swine/Shandong/S113/2014|H1N1|China
A/swine/Hebei/0221/2017|H1N1|China
A/swine/Shandong/1207/2016|H1N1|China
A/swine/Heilongjiang/0140/2017|H1N1|China
A/swine/Shandong/1203/2016|H1N1|China
A/swine/Shandong/0334/2017|H1N1|China
A/swine/Heilongjiang/0110/2017|H1N1|China
A/swine/Hebei/0116/2017|H1N1|China
A/swine/Korea/0138/2022|H1N1
A/swine/Korea/0139/2022|H1N1
A/swine/Korea/0629/2021|H1N1
A/swine/Korea/0119−3/2022|H1N1
A/swine/Korea/0119−4/2022|H1N1
A/swine/Korea/0119−1/2022|H1N1
A/swine/Korea/0118−1/2022|H1N1
A/swine/Korea/0760/2021|H1N1
A/swine/Korea/0878/2021|H1N1
A/swine/Korea/0119−2/2022|H1N1
A/swine/Korea/0118−2/2022|H1N1
A/Mexico/4486/2009|H1N1|Mexico
A/Sichuan/1/2009|H1N1|China
A/California/07/2009|H1N1|USA
A/swine/Korea/CY01_04/2012|H1N1|South_Korea
A/swine/Korea/CY01_05/2012|H1N1|South_Korea
A/swine/Korea/CY11_02/2011|H1N1|South_Korea
A/swine/Korea/CY11_01/2011|H1N1|South_Korea
A/swine/Korea/CY01_06/2012|H1N1|South_Korea
A/Korea/01/2009|H1N1|South_Korea
A/swine/Korea/SCJ06/2009|H1N1|South_Korea
A/swine/Korea/SCJ01/2009|H1N1|South_Korea
A/swine/Korea/SCJ02/2009|H1N1|South_Korea
A/swine/Korea/SCJ04/2009|H1N1|South_Korea
A/swine/Korea/SCJ03/2009|H1N1|South_Korea
A/swine/Korea/SCJ05/2009|H1N1|South_Korea
A/swine/Korea/SCJ07/2009|H1N1|South_Korea
A/swine/Korea/SCJ08/2009|H1N1|South_Korea
A/Zhejiang_Yiwu/11/2009|H1N1|China
A/Wisconsin/629_D00160/2009|H1N1|USA
A/Korea/NAP_1/2009|H1N1|South_Korea
A/Korea/CJ63/2009|H1N1|South_Korea
A/swine/Korea/VD01/2009|H1N1|South_Korea
A/New_York/18/2009|H1N1|USA
A/Korea/CJ04/2009|H1N1|South_Korea
A/Korea/CJ40/2009|H1N1|South_Korea
A/swine/Korea/SCJ20/2009|H1N1|South_Korea
A/swine/Korea/SCJ26/2009|H1N1|South_Korea
A/swine/Korea/SCJ28/2010|H1N1|South_Korea
A/swine/Korea/SCJ33/2010|H1N1|South_Korea
A/swine/Korea/SCJ13/2009|H1N1|South_Korea
A/swine/Korea/SCJ11/2009|H1N1|South_Korea
A/swine/Korea/SCJ12/2009|H1N1|South_Korea
A/swine/Korea/SCJ41/2010|H1N1|South_Korea
A/Taiwan/143/2009|H1N1|Taiwan
A/Korea/CJ68/2009|H1N1|South_Korea
A/swine/Korea/GBCG01/2010|H1N1|South_Korea
A/swine/Korea/SCJ09/2009|H1N1|South_Korea
A/swine/Korea/SCJ10/2009|H1N1|South_Korea
A/Karasuk/01/2010|H1N1|Russia
A/Gangwon/61/2016|H1N1|South_Korea
A/Chungnam/29/2016|H1N1|South_Korea
A/Seoul/224/2016|H1N1|South_Korea
A/Chungbuk/106/2016|H1N1|South_Korea
A/Jeju/759/2017|H1N1|South_Korea
A/Gwangju/766/2017|H1N1|South_Korea
A/Daejeon/1455/2018|H1N1|South_Korea
A/Daegu/1454/2018|H1N1|South_Korea
A/South_Korea/9706/2019|H1N1|South_Korea
A/South_Korea/9115/2019|H1N1|South_Korea
A/South_Korea/9117/2019|H1N1|South_Korea
A/swine/SouthKorea/BRI5/2020|H1N1|South_Korea
A/swine/Korea/0171/2022|H1N1
A/South_Korea/7536/2018|H1N1|South_Korea
A/South_Korea/7880/2018|H1N1|South_Korea
A/South_Korea/7871/2018|H1N1|South_Korea
0.05
Lineage
A(H1N1)pdm09
Classical Swine
Eurasian Avian
Eurasian Avian−like Swine
Human seasonal H1N1 (pre−2009)
North American Avian
Host
Avian
Human
Swine
Country
Canada
USA
Mexico
Japan
Korea
China
Mongolia
Taiwan
New_Zealand
Georgia
Germany
Italy
Netherlands
Spain
Russia

NA (N2)

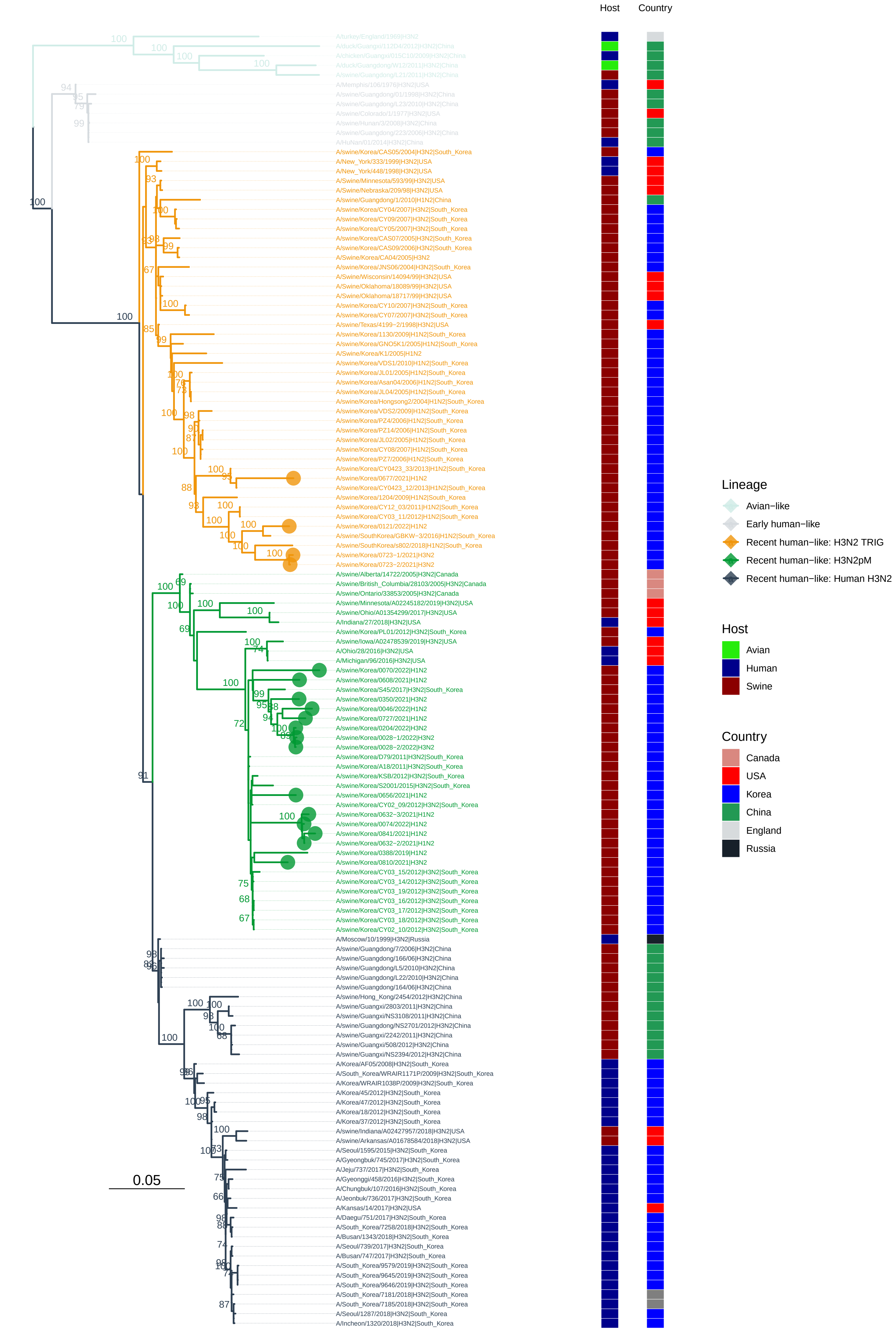

Host
Country
A/turkey/England/1969|H3N2
A/duck/Guangxi/112D4/2012|H3N2|China
A/chicken/Guangxi/015C10/2009|H3N2|China
A/duck/Guangdong/W12/2011|H3N2|China
A/swine/Guangdong/L21/2011|H3N2|China
A/Memphis/106/1976|H3N2|USA
A/swine/Guangdong/01/1998|H3N2|China
A/swine/Guangdong/L23/2010|H3N2|China
A/swine/Colorado/1/1977|H3N2|USA
A/swine/Hunan/3/2008|H3N2|China
A/swine/Guangdong/223/2006|H3N2|China
A/HuNan/01/2014|H3N2|China
A/swine/Korea/CAS05/2004|H3N2|South_Korea
A/New_York/333/1999|H3N2|USA
A/New_York/448/1998|H3N2|USA
A/Swine/Minnesota/593/99|H3N2|USA
A/Swine/Nebraska/209/98|H3N2|USA
A/swine/Guangdong/1/2010|H1N2|China
A/swine/Korea/CY04/2007|H3N2|South_Korea
A/swine/Korea/CY09/2007|H3N2|South_Korea
A/swine/Korea/CY05/2007|H3N2|South_Korea
A/swine/Korea/CAS07/2005|H3N2|South_Korea
A/swine/Korea/CAS09/2006|H3N2|South_Korea
A/Swine/Korea/CA04/2005|H3N2
A/swine/Korea/JNS06/2004|H3N2|South_Korea
A/Swine/Wisconsin/14094/99|H3N2|USA
A/Swine/Oklahoma/18089/99|H3N2|USA
A/Swine/Oklahoma/18717/99|H3N2|USA
A/swine/Korea/CY10/2007|H3N2|South_Korea
A/swine/Korea/CY07/2007|H3N2|South_Korea
A/Swine/Texas/4199−2/1998|H3N2|USA
A/swine/Korea/1130/2009|H1N2|South_Korea
A/swine/Korea/GNO5K1/2005|H1N2|South_Korea
A/Swine/Korea/K1/2005|H1N2
A/swine/Korea/VDS1/2010|H1N2|South_Korea
A/swine/Korea/JL01/2005|H1N2|South_Korea
A/swine/Korea/Asan04/2006|H1N2|South_Korea
A/swine/Korea/JL04/2005|H1N2|South_Korea
A/swine/Korea/Hongsong2/2004|H1N2|South_Korea
A/swine/Korea/VDS2/2009|H1N2|South_Korea
A/swine/Korea/PZ4/2006|H1N2|South_Korea
A/swine/Korea/PZ14/2006|H1N2|South_Korea
A/swine/Korea/JL02/2005|H1N2|South_Korea
A/swine/Korea/CY08/2007|H1N2|South_Korea
A/swine/Korea/PZ7/2006|H1N2|South_Korea
A/swine/Korea/CY0423_33/2013|H1N2|South_Korea
A/swine/Korea/0677/2021|H1N2
A/swine/Korea/CY0423_12/2013|H1N2|South_Korea
A/swine/Korea/1204/2009|H1N2|South_Korea
A/swine/Korea/CY12_03/2011|H1N2|South_Korea
A/swine/Korea/CY03_11/2012|H1N2|South_Korea
A/swine/Korea/0121/2022|H1N2
A/swine/SouthKorea/GBKW−3/2016|H1N2|South_Korea
A/swine/SouthKorea/s802/2018|H1N2|South_Korea
A/swine/Korea/0723−1/2021|H3N2
A/swine/Korea/0723−2/2021|H3N2
A/swine/Alberta/14722/2005|H3N2|Canada
A/swine/British_Columbia/28103/2005|H3N2|Canada
A/swine/Ontario/33853/2005|H3N2|Canada
A/swine/Minnesota/A02245182/2019|H3N2|USA
A/swine/Ohio/A01354299/2017|H3N2|USA
A/Indiana/27/2018|H3N2|USA
A/swine/Korea/PL01/2012|H3N2|South_Korea
A/swine/Iowa/A02478539/2019|H3N2|USA
A/Ohio/28/2016|H3N2|USA
A/Michigan/96/2016|H3N2|USA
A/swine/Korea/0070/2022|H1N2
A/swine/Korea/0608/2021|H1N2
A/swine/Korea/S45/2017|H3N2|South_Korea
A/swine/Korea/0350/2021|H3N2
A/swine/Korea/0046/2022|H1N2
A/swine/Korea/0727/2021|H1N2
A/swine/Korea/0204/2022|H3N2
A/swine/Korea/0028−1/2022|H3N2
A/swine/Korea/0028−2/2022|H3N2
A/swine/Korea/D79/2011|H3N2|South_Korea
A/swine/Korea/A18/2011|H3N2|South_Korea
A/swine/Korea/KSB/2012|H3N2|South_Korea
A/swine/Korea/S2001/2015|H3N2|South_Korea
A/swine/Korea/0656/2021|H1N2
A/swine/Korea/CY02_09/2012|H3N2|South_Korea
A/swine/Korea/0632−3/2021|H1N2
A/swine/Korea/0074/2022|H1N2
A/swine/Korea/0841/2021|H1N2
A/swine/Korea/0632−2/2021|H1N2
A/swine/Korea/0388/2019|H1N2
A/swine/Korea/0810/2021|H3N2
A/swine/Korea/CY03_15/2012|H3N2|South_Korea
A/swine/Korea/CY03_14/2012|H3N2|South_Korea
A/swine/Korea/CY03_19/2012|H3N2|South_Korea
A/swine/Korea/CY03_16/2012|H3N2|South_Korea
A/swine/Korea/CY03_17/2012|H3N2|South_Korea
A/swine/Korea/CY03_18/2012|H3N2|South_Korea
A/swine/Korea/CY02_10/2012|H3N2|South_Korea
A/Moscow/10/1999|H3N2|Russia
A/swine/Guangdong/7/2006|H3N2|China
A/swine/Guangdong/166/06|H3N2|China
A/swine/Guangdong/L5/2010|H3N2|China
A/swine/Guangdong/L22/2010|H3N2|China
A/swine/Guangdong/164/06|H3N2|China
A/swine/Hong_Kong/2454/2012|H3N2|China
A/swine/Guangxi/2803/2011|H3N2|China
A/swine/Guangxi/NS3108/2011|H3N2|China
A/swine/Guangdong/NS2701/2012|H3N2|China
A/swine/Guangxi/2242/2011|H3N2|China
A/swine/Guangxi/508/2012|H3N2|China
A/swine/Guangxi/NS2394/2012|H3N2|China
A/Korea/AF05/2008|H3N2|South_Korea
A/South_Korea/WRAIR1171P/2009|H3N2|South_Korea
A/Korea/WRAIR1038P/2009|H3N2|South_Korea
A/Korea/45/2012|H3N2|South_Korea
A/Korea/47/2012|H3N2|South_Korea
A/Korea/18/2012|H3N2|South_Korea
A/Korea/37/2012|H3N2|South_Korea
A/swine/Indiana/A02427957/2018|H3N2|USA
A/swine/Arkansas/A01678584/2018|H3N2|USA
A/Seoul/1595/2015|H3N2|South_Korea
A/Gyeongbuk/745/2017|H3N2|South_Korea
A/Jeju/737/2017|H3N2|South_Korea
A/Gyeonggi/458/2016|H3N2|South_Korea
A/Chungbuk/107/2016|H3N2|South_Korea
A/Jeonbuk/736/2017|H3N2|South_Korea
A/Kansas/14/2017|H3N2|USA
A/Daegu/751/2017|H3N2|South_Korea
A/South_Korea/7258/2018|H3N2|South_Korea
A/Busan/1343/2018|H3N2|South_Korea
A/Seoul/739/2017|H3N2|South_Korea
A/Busan/747/2017|H3N2|South_Korea
A/South_Korea/9579/2019|H3N2|South_Korea
A/South_Korea/9645/2019|H3N2|South_Korea
A/South_Korea/9646/2019|H3N2|South_Korea
A/South_Korea/7181/2018|H3N2|South_Korea
A/South_Korea/7185/2018|H3N2|South_Korea
A/Seoul/1287/2018|H3N2|South_Korea
A/Incheon/1320/2018|H3N2|South_Korea
0.05
Lineage
Avian−like
Early human−like
Recent human−like: H3N2 TRIG
Recent human−like: H3N2pM
Recent human−like: Human H3N2
Host
Avian
Human
Swine
Country
Canada
USA
Korea
China
England
Russia

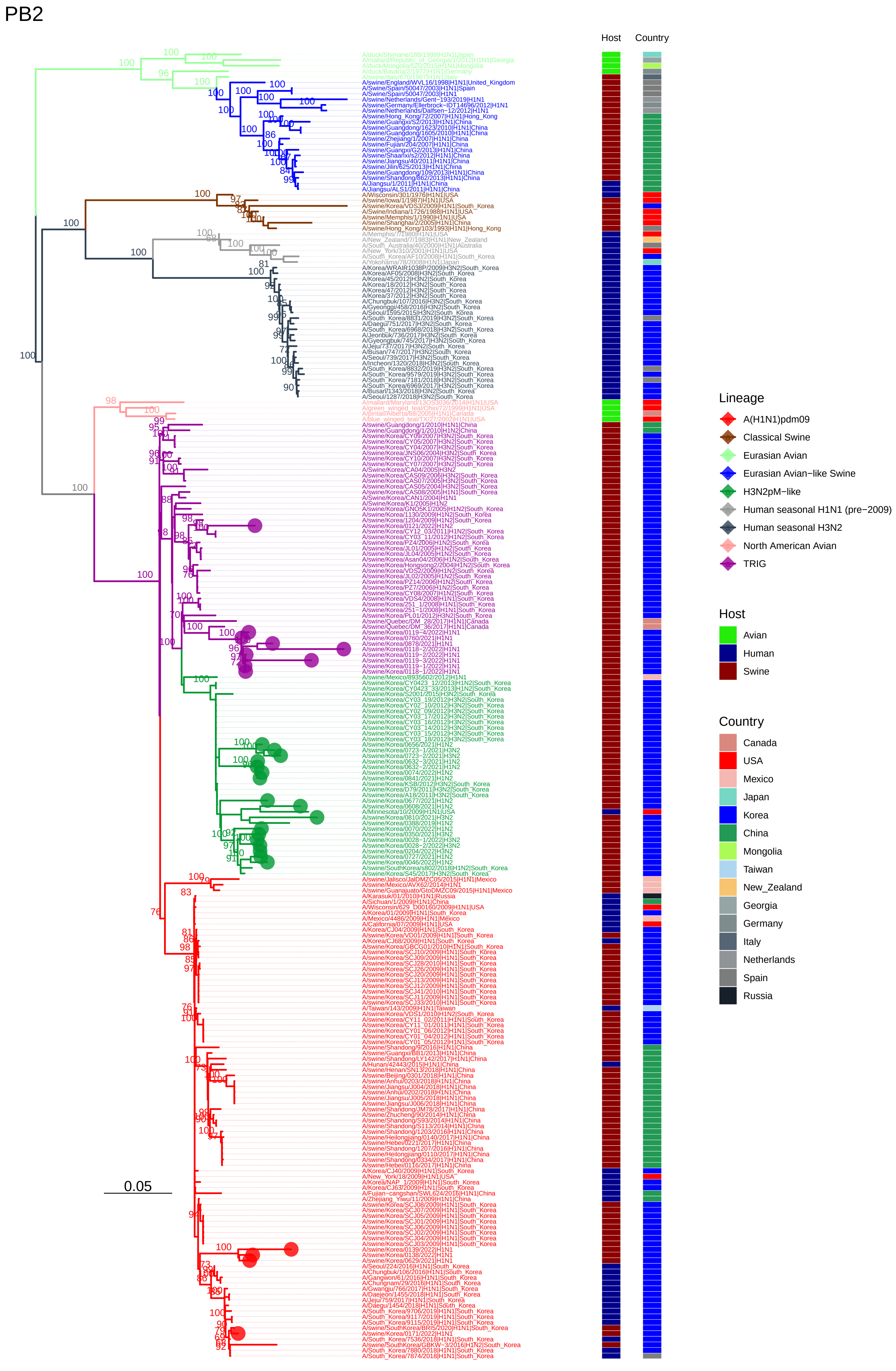

PB2
Host
Country
Lineage
A(H1N1)pdm09
Classical Swine
Eurasian Avian
Eurasian Avian−like Swine
H3N2pM−like
Human seasonal H1N1 (pre−2009)
Human seasonal H3N2
North American Avian
TRIG
Host
Avian
Human
Swine
Country
Canada
USA
Mexico
Japan
Korea
China
Mongolia
Taiwan
New_Zealand
Georgia
Germany
Italy
Netherlands
Spain
Russia
0.05

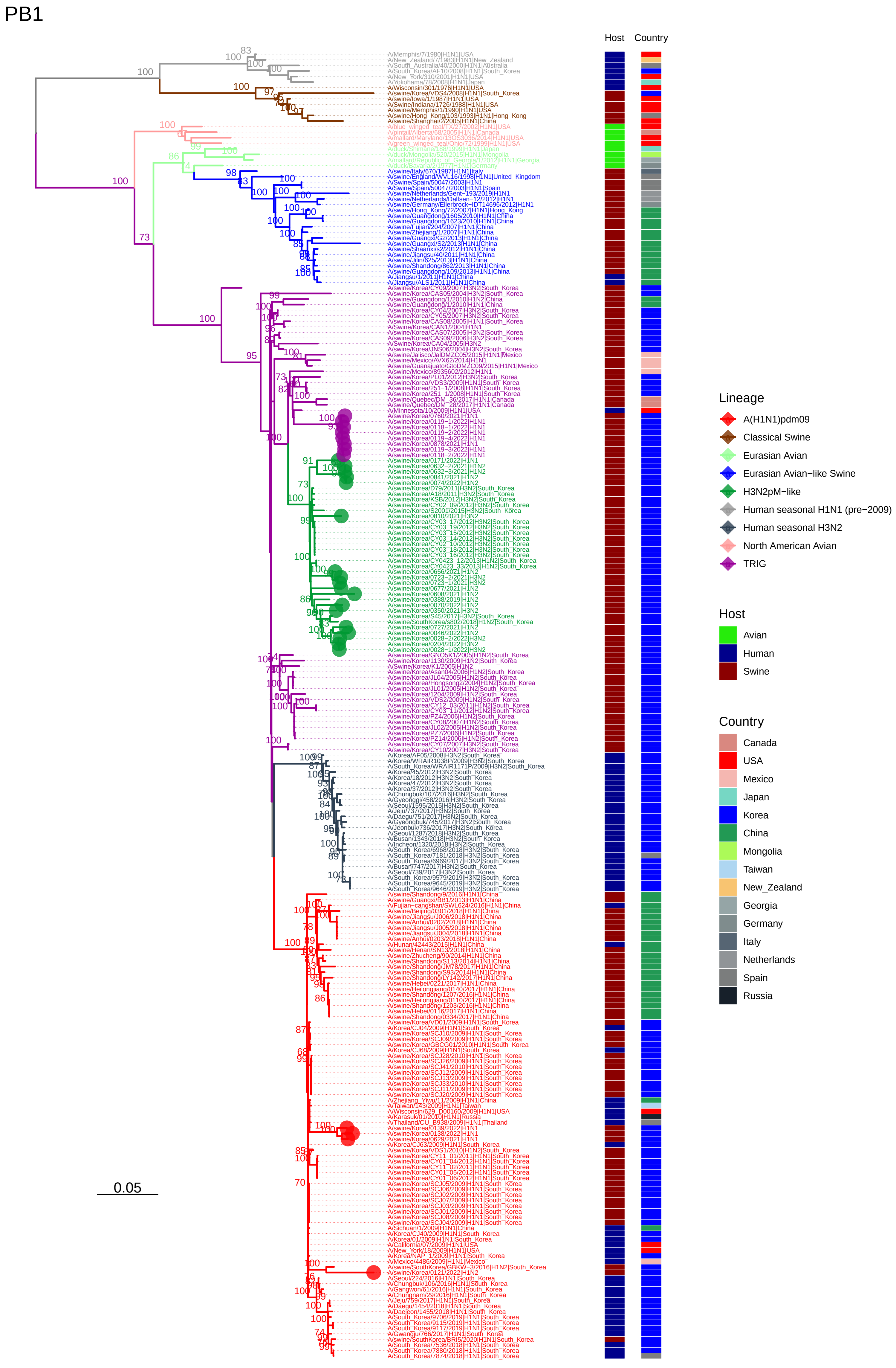
PB1
Host
Country
Lineage
A(H1N1)pdm09
Classical Swine
Eurasian Avian
Eurasian Avian−like Swine
H3N2pM−like
Human seasonal H1N1 (pre−2009)
Human seasonal H3N2
North American Avian
TRIG
Host
Avian
Human
Swine
Country
Canada
USA
Mexico
Japan
Korea
China
Mongolia
Taiwan
New_Zealand
Georgia
Germany
Italy
Netherlands
Spain
Russia
0.05

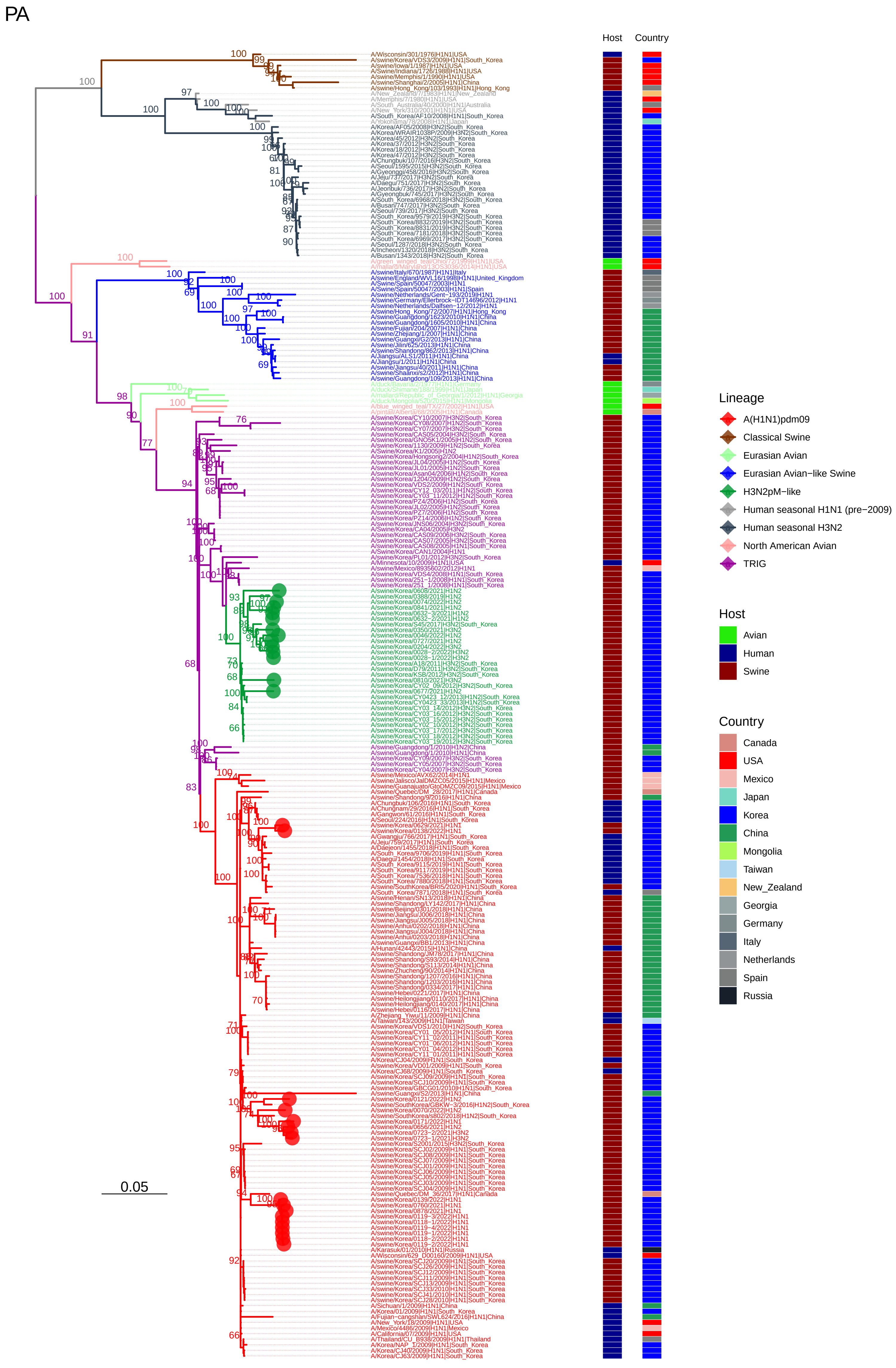
PA
Host
Country
A/Wisconsin/301/1976|H1N1|USA
A/swine/Korea/VDS3/2009|H1N1|South_Korea
A/swine/Iowa/1/1987|H1N1|USA
A/Swine/Indiana/1726/1988|H1N1|USA
A/swine/Memphis/1/1990|H1N1|USA
A/swine/Shanghai/2/2005|H1N1|China
A/swine/Hong_Kong/103/1993|H1N1|Hong_Kong
A/New_Zealand/7/1983|H1N1|New_Zealand
A/Memphis/7/1980|H1N1|USA
A/South_Australia/40/2000|H1N1|Australia
A/New_York/310/2001|H1N1|USA
A/South_Korea/AF10/2008|H1N1|South_Korea
A/Yokohama/78/2008|H1N1|Japan
A/Korea/AF05/2008|H3N2|South_Korea
A/Korea/WRAIR1038P/2009|H3N2|South_Korea
A/Korea/45/2012|H3N2|South_Korea
A/Korea/37/2012|H3N2|South_Korea
A/Korea/18/2012|H3N2|South_Korea
A/Korea/47/2012|H3N2|South_Korea
A/Chungbuk/107/2016|H3N2|South_Korea
A/Seoul/1595/2015|H3N2|South_Korea
A/Gyeonggi/458/2016|H3N2|South_Korea
A/Jeju/737/2017|H3N2|South_Korea
A/Daegu/751/2017|H3N2|South_Korea
A/Jeonbuk/736/2017|H3N2|South_Korea
A/Gyeongbuk/745/2017|H3N2|South_Korea
A/South_Korea/6968/2018|H3N2|South_Korea
A/Busan/747/2017|H3N2|South_Korea
A/Seoul/739/2017|H3N2|South_Korea
A/South_Korea/9579/2019|H3N2|South_Korea
A/South_Korea/8832/2019|H3N2|South_Korea
A/South_Korea/8831/2019|H3N2|South_Korea
A/South_Korea/7181/2018|H3N2|South_Korea
A/South_Korea/6969/2017|H3N2|South_Korea
A/Seoul/1287/2018|H3N2|South_Korea
A/Incheon/1320/2018|H3N2|South_Korea
A/Busan/1343/2018|H3N2|South_Korea
A/green_winged_teal/Ohio/72/1999|H1N1|USA
A/mallard/Maryland/13OS3036/2014|H1N1|USA
A/swine/Italy/670/1987|H1N1|Italy
A/swine/England/WVL16/1998|H1N1|United_Kingdom
A/Swine/Spain/50047/2003|H1N1
A/Swine/Spain/50047/2003|H1N1|Spain
A/swine/Netherlands/Gent-193/2019|H1N1
A/swine/Germany/Ellerbrock-IDT14696/2012|H1N1
A/swine/Netherlands/Dalfsen-12/2012|H1N1
A/swine/Hong_Kong/72/2007|H1N1|Hong_Kong
A/swine/Guangdong/1623/2010|H1N1|China
A/swine/Guangdong/1605/2010|H1N1|China
A/swine/Fujian/204/2007|H1N1|China
A/swine/Zhejiang/1/2007|H1N1|China
A/swine/Guangxi/G2/2013|H1N1|China
A/swine/Jilin/625/2013|H1N1|China
A/swine/Shandong/862/2013|H1N1|China
A/Jiangsu/ALS1/2011|H1N1|China
A/Jiangsu/1/2011|H1N1|China
A/swine/Jiangsu/40/2011|H1N1|China
A/swine/Shaanxi/s2/2012|H1N1|China
A/swine/Guangdong/109/2013|H1N1|China
A/duck/Bavaria/2/1977|H1N1|Germany
A/duck/Shimane/188/1999|H1N1|Japan
A/mallard/Republic_of_Georgia/1/2012|H1N1|Georgia
A/duck/Mongolia/520/2015|H1N1|Mongolia
A/blue_winged_teal/TX/27/2002|H1N1|USA
A/pintail/Alberta/68/2005|H1N1|Canada
A/swine/Korea/CY10/2007|H3N2|South_Korea
A/swine/Korea/CY08/2007|H1N2|South_Korea
A/swine/Korea/CY07/2007|H1N2|South_Korea
A/swine/Korea/CAS05/2004|H3N2|South_Korea
A/swine/Korea/GNO5K1/2005|H1N2|South_Korea
A/swine/Korea/1130/2009|H1N2|South_Korea
A/Swine/Korea/K1/2005|H1N2
A/swine/Korea/Hongsong2/2004|H1N2|South_Korea
A/swine/Korea/JL04/2005|H1N2|South_Korea
A/swine/Korea/JL01/2005|H1N2|South_Korea
A/swine/Korea/Asan04/2006|H1N2|South_Korea
A/swine/Korea/1204/2009|H1N2|South_Korea
A/swine/Korea/VDS2/2009|H1N2|South_Korea
A/swine/Korea/CY12_03/2011|H1N2|South_Korea
A/swine/Korea/CY03_11/2012|H1N2|South_Korea
A/swine/Korea/PZ4/2006|H1N2|South_Korea
A/swine/Korea/JL02/2005|H1N2|South_Korea
A/swine/Korea/PZ7/2006|H1N2|South_Korea
A/swine/Korea/PZ14/2006|H1N2|South_Korea
A/swine/Korea/JNS06/2004|H1N2|South_Korea
A/Swine/Korea/CA04/2005|H3N2
A/swine/Korea/CAS09/2006|H3N2|South_Korea
A/swine/Korea/CAS07/2005|H3N2|South_Korea
A/swine/Korea/CAS08/2005|H3N2|South_Korea
A/Swine/Korea/CAN1/2004|H1N1
A/swine/Korea/PL01/2012|H3N2|South_Korea
A/Minnesota/10/2009|H1N1|USA
A/swine/Mexico/8935602/2012|H1N1
A/swine/Korea/VDS4/2008|H1N1|South_Korea
A/swine/Korea/251-1/2008|H1N1|South_Korea
A/swine/Korea/251_1/2008|H1N1|South_Korea
A/swine/Korea/0608/2021|H1N2
A/swine/Korea/0388/2019|H1N2
A/swine/Korea/0074/2022|H1N2
A/swine/Korea/0841/2021|H1N2
A/swine/Korea/0632-3/2021|H1N2
A/swine/Korea/0632-2/2021|H1N2
A/swine/Korea/S45/2017|H3N2|South_Korea
A/swine/Korea/0350/2021|H3N2
A/swine/Korea/0046/2022|H1N2
A/swine/Korea/0727/2021|H1N2
A/swine/Korea/0204/2022|H3N2
A/swine/Korea/0028-2/2022|H3N2
A/swine/Korea/0028-1/2022|H3N2
A/swine/Korea/A18/2011|H3N2|South_Korea
A/swine/Korea/D79/2011|H3N2|South_Korea
A/swine/Korea/KSB/2012|H3N2|South_Korea
A/swine/Korea/0810/2021|H3N2
A/swine/Korea/CY02_09/2012|H3N2|South_Korea
A/swine/Korea/0677/2021|H1N2
A/swine/Korea/CY0423_12/2013|H1N2|South_Korea
A/swine/Korea/CY0423_33/2013|H1N2|South_Korea
A/swine/Korea/CY03_14/2012|H3N2|South_Korea
A/swine/Korea/CY03_16/2012|H3N2|South_Korea
A/swine/Korea/CY03_15/2012|H3N2|South_Korea
A/swine/Korea/CY02_10/2012|H3N2|South_Korea
A/swine/Korea/CY03_17/2012|H3N2|South_Korea
A/swine/Korea/CY03_18/2012|H3N2|South_Korea
A/swine/Korea/CY03_19/2012|H3N2|South_Korea
A/swine/Guangdong/1/2010|H1N2|China
A/swine/Guangdong/1/2010|H1N1|China
A/swine/Korea/CY09/2007|H3N2|South_Korea
A/swine/Korea/CY05/2007|H3N2|South_Korea
A/swine/Korea/CY04/2007|H3N2|South_Korea
A/swine/Mexico/AVX62/2014|H1N1
A/swine/Jalisco/JalDMZC05/2015|H1N1|Mexico
A/swine/Guanajuato/GtoDMZC09/2015|H1N1|Mexico
A/swine/Quebec/DM_28/2017|H1N1|Canada
A/swine/Shandong/9/2016|H1N1|China
A/Chungbuk/106/2016|H1N1|South_Korea
A/Chungnam/29/2016|H1N1|South_Korea
A/Gangwon/61/2016|H1N1|South_Korea
A/Seoul/224/2016|H1N1|South_Korea
A/swine/Korea/0629/2021|H1N1
A/swine/Korea/0138/2022|H1N1
A/Gwangju/766/2017|H1N1|South_Korea
A/Jeju/759/2017|H1N1|South_Korea
A/Daejeon/1455/2018|H1N1|South_Korea
A/South_Korea/9706/2019|H1N1|South_Korea
A/Daegu/1454/2018|H1N1|South_Korea
A/South_Korea/9115/2019|H1N1|South_Korea
A/South_Korea/9117/2019|H1N1|South_Korea
A/South_Korea/7536/2018|H1N1|South_Korea
A/South_Korea/7880/2018|H1N1|South_Korea
A/swine/SouthKorea/BRI5/2020|H1N1|South_Korea
A/South_Korea/7871/2018|H1N1|South_Korea
A/swine/Henan/SN13/2018|H1N1|China
A/swine/Shandong/LY142/2017|H1N1|China
A/swine/Beijing/0301/2018|H1N1|China
A/swine/Jiangsu/J006/2018|H1N1|China
A/swine/Jiangsu/J005/2018|H1N1|China
A/swine/Anhui/0202/2018|H1N1|China
A/swine/Jiangsu/J004/2018|H1N1|China
A/swine/Anhui/0203/2018|H1N1|China
A/swine/Guangxi/BB1/2013|H1N1|China
A/Hunan/42443/2015|H1N1|China
A/swine/Shandong/JM78/2017|H1N1|China
A/swine/Shandong/S93/2014|H1N1|China
A/swine/Shandong/S113/2014|H1N1|China
A/swine/Zhucheng/90/2014|H1N1|China
A/swine/Shandong/1207/2016|H1N1|China
A/swine/Shandong/1203/2016|H1N1|China
A/swine/Shandong/0334/2017|H1N1|China
A/swine/Hebei/0221/2017|H1N1|China
A/swine/Heilongjiang/0110/2017|H1N1|China
A/swine/Heilongjiang/0140/2017|H1N1|China
A/swine/Hebei/0116/2017|H1N1|China
A/Zhejiang_Yiwu/11/2009|H1N1|China
A/Taiwan/143/2009|H1N1|Taiwan
A/swine/Korea/VDS1/2010|H1N2|South_Korea
A/swine/Korea/CY01_05/2012|H1N1|South_Korea
A/swine/Korea/CY11_02/2011|H1N1|South_Korea
A/swine/Korea/CY01_06/2012|H1N1|South_Korea
A/swine/Korea/CY01_04/2012|H1N1|South_Korea
A/swine/Korea/CY11_01/2011|H1N1|South_Korea
A/Korea/CJ04/2009|H1N1|South_Korea
A/swine/Korea/VD01/2009|H1N1|South_Korea
A/Korea/CJ68/2009|H1N1|South_Korea
A/swine/Korea/SCJ09/2009|H1N1|South_Korea
A/swine/Korea/SCJ10/2009|H1N1|South_Korea
A/swine/Korea/GBCG01/2010|H1N1|South_Korea
A/swine/Guangxi/S2/2013|H1N1|China
A/swine/Korea/0121/2022|H1N2
A/swine/SouthKorea/GBKW-3/2016|H1N2|South_Korea
A/swine/Korea/0070/2022|H1N2
A/swine/SouthKorea/s802/2018|H1N2|South_Korea
A/swine/Korea/0171/2022|H1N1
A/swine/Korea/0656/2021|H1N2
A/swine/Korea/0723-2/2021|H3N2
A/swine/Korea/0723-1/2021|H3N2
A/swine/Korea/S2001/2015|H3N2|South_Korea
A/swine/Korea/SCJ02/2009|H1N1|South_Korea
A/swine/Korea/SCJ08/2009|H1N1|South_Korea
A/swine/Korea/SCJ07/2009|H1N1|South_Korea
A/swine/Korea/SCJ01/2009|H1N1|South_Korea
A/swine/Korea/SCJ06/2009|H1N1|South_Korea
A/swine/Korea/SCJ05/2009|H1N1|South_Korea
A/swine/Korea/SCJ03/2009|H1N1|South_Korea
A/swine/Korea/SCJ04/2009|H1N1|South_Korea
A/swine/Quebec/DM_36/2017|H1N1|Canada
A/swine/Korea/0139/2022|H1N1
A/swine/Korea/0760/2021|H1N1
A/swine/Korea/0878/2021|H1N1
A/swine/Korea/0119-3/2022|H1N1
A/swine/Korea/0118-1/2022|H1N1
A/swine/Korea/0119-4/2022|H1N1
A/swine/Korea/0119-1/2022|H1N1
A/swine/Korea/0118-2/2022|H1N1
A/swine/Korea/0119-2/2022|H1N1
A/Karasuk/01/2010|H1N1|Russia
A/Wisconsin/629_D00160/2009|H1N1|USA
A/swine/Korea/SCJ20/2009|H1N1|South_Korea
A/swine/Korea/SCJ26/2009|H1N1|South_Korea
A/swine/Korea/SCJ12/2009|H1N1|South_Korea
A/swine/Korea/SCJ11/2009|H1N1|South_Korea
A/swine/Korea/SCJ13/2009|H1N1|South_Korea
A/swine/Korea/SCJ33/2010|H1N1|South_Korea
A/swine/Korea/SCJ41/2010|H1N1|South_Korea
A/swine/Korea/SCJ28/2010|H1N1|South_Korea
A/Sichuan/1/2009|H1N1|China
A/Korea/01/2009|H1N1|South_Korea
A/Fujian-cangshan/SWL624/2016|H1N1|China
A/New_York/18/2009|H1N1|USA
A/Mexico/4486/2009|H1N1|Mexico
A/California/07/2009|H1N1|USA
A/Thailand/CU_B938/2009|H1N1|Thailand
A/Korea/NAP_1/2009|H1N1|South_Korea
A/Korea/CJ40/2009|H1N1|South_Korea
A/Korea/CJ63/2009|H1N1|South_Korea
0.05
Lineage
A(H1N1)pdm09
Classical Swine
Eurasian Avian
Eurasian Avian−like Swine
H3N2pM−like
Human seasonal H1N1 (pre−2009)
Human seasonal H3N2
North American Avian
TRIG
Host
Avian
Human
Swine
Country
Canada
USA
Mexico
Japan
Korea
China
Mongolia
Taiwan
New_Zealand
Georgia
Germany
Italy
Netherlands
Spain
Russia

NP

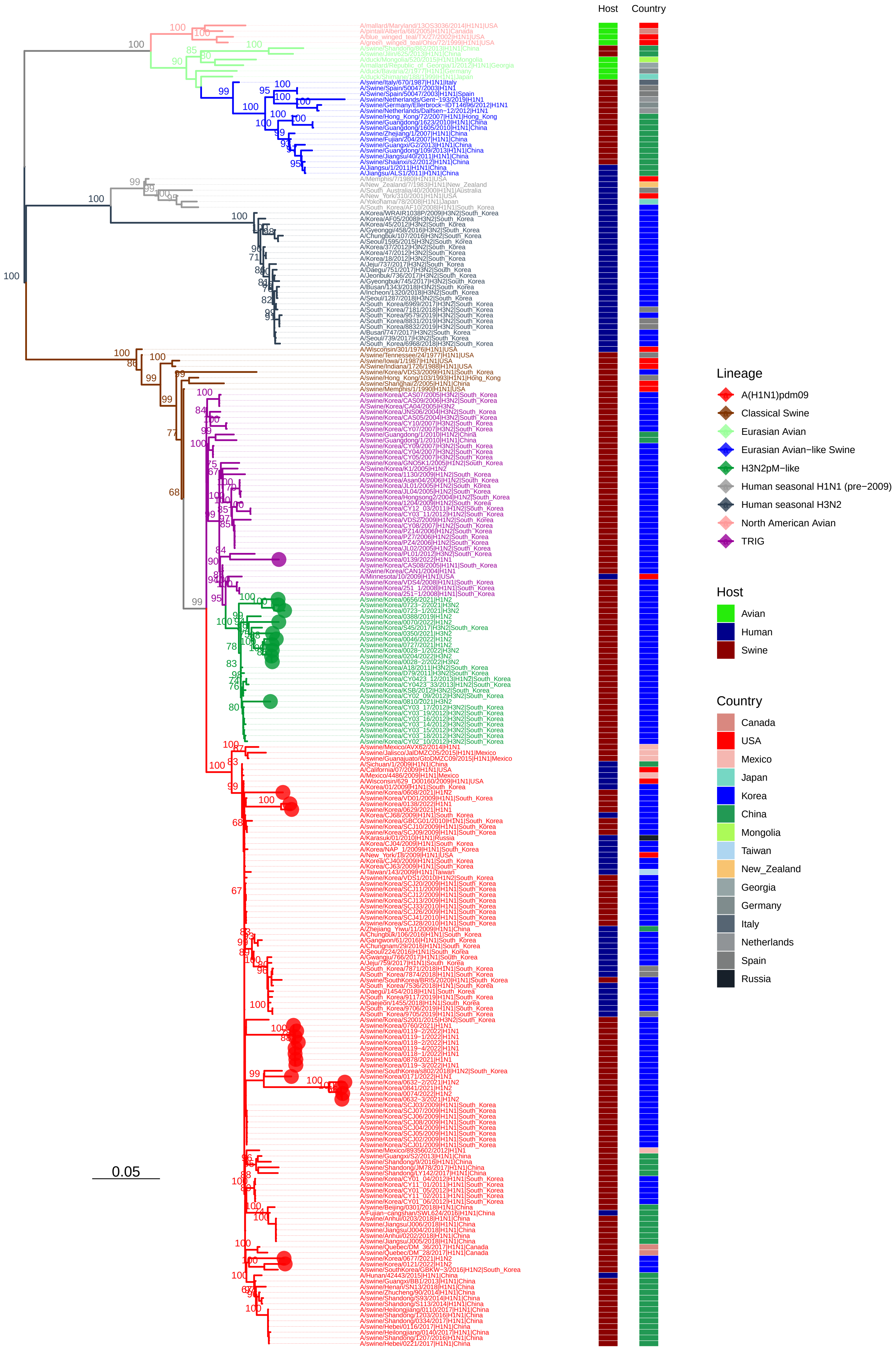
Host
Country
0.05
Lineage
A(H1N1)pdm09
Classical Swine
Eurasian Avian
Eurasian Avian−like Swine
H3N2pM−like
Human seasonal H1N1 (pre−2009)
Human seasonal H3N2
North American Avian
TRIG
Host
Avian
Human
Swine
Country
Canada
USA
Mexico
Japan
Korea
China
Mongolia
Taiwan
New_Zealand
Georgia
Germany
Italy
Netherlands
Spain
Russia

M

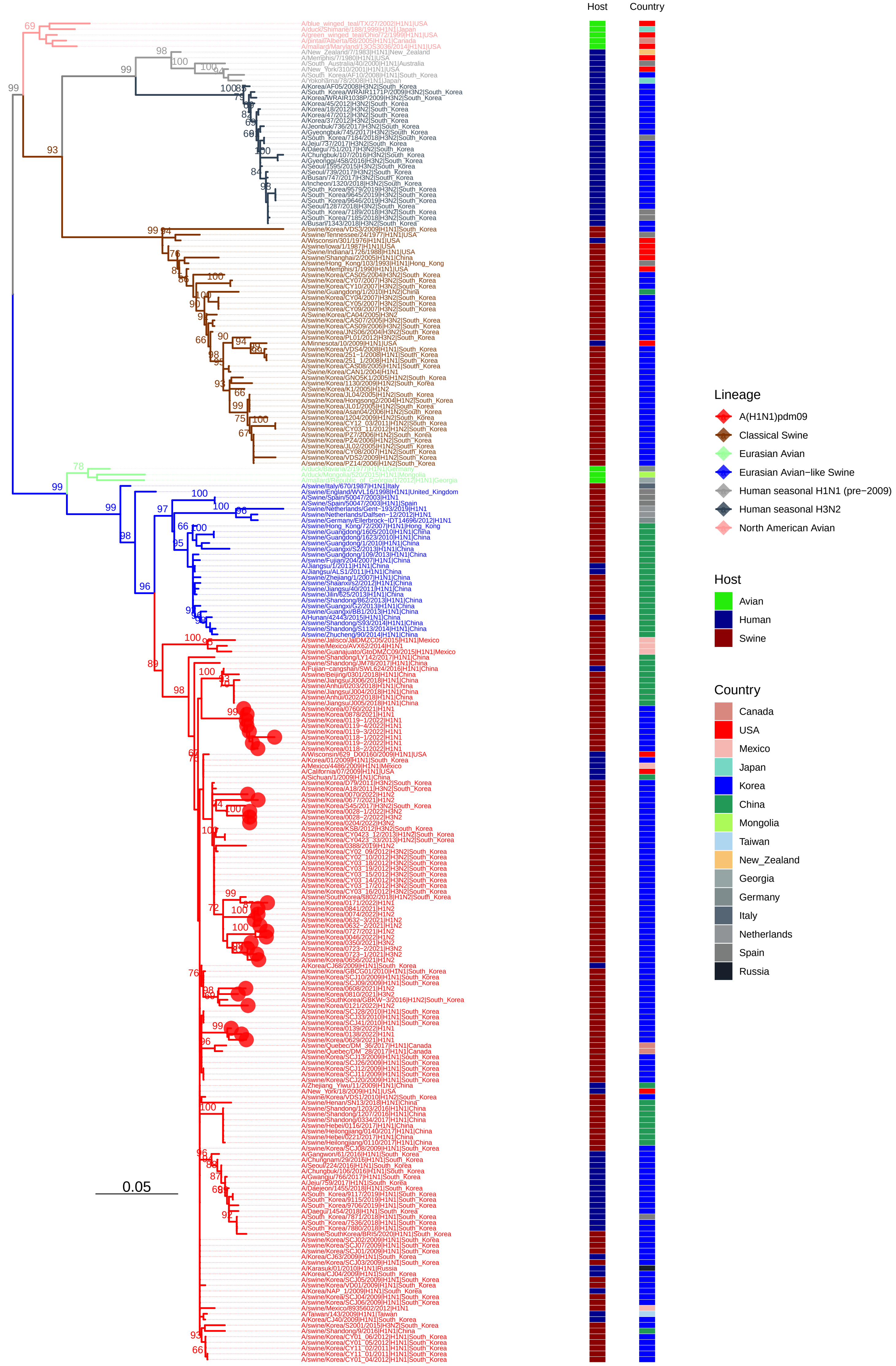
Host
Country
0.05
Lineage
A(H1N1)pdm09
Classical Swine
Eurasian Avian
Eurasian Avian−like Swine
Human seasonal H1N1 (pre−2009)
Human seasonal H3N2
North American Avian
Host
Avian
Human
Swine
Country
Canada
USA
Mexico
Japan
Korea
China
Mongolia
Taiwan
New_Zealand
Georgia
Germany
Italy
Netherlands
Spain
Russia

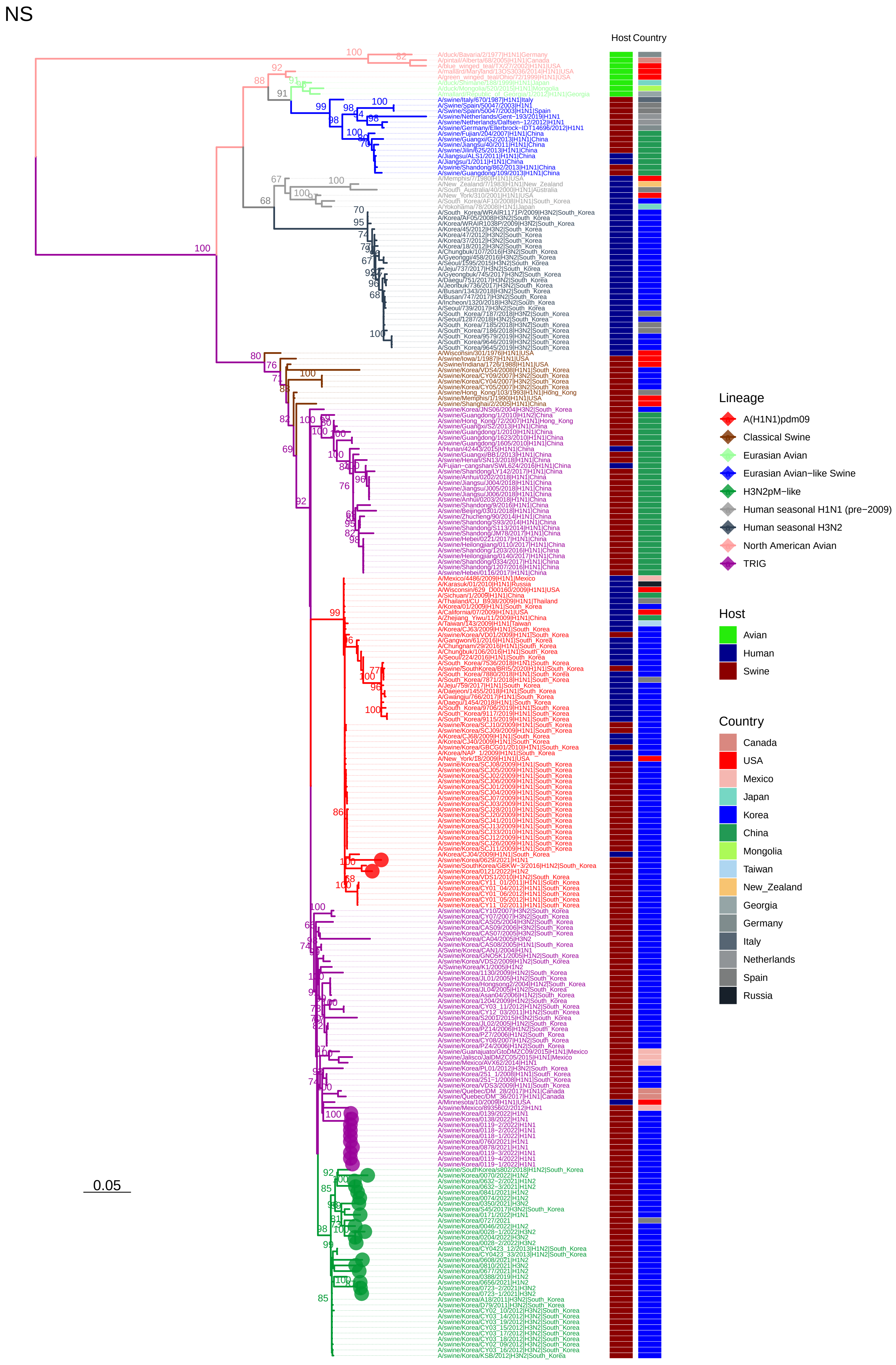

NS
Host Country
Lineage
A(H1N1)pdm09
Classical Swine
Eurasian Avian
Eurasian Avian−like Swine
H3N2pM−like
Human seasonal H1N1 (pre−2009)
Human seasonal H3N2
North American Avian
TRIG
Host
Avian
Human
Swine
Country
Canada
USA
Mexico
Japan
Korea
China
Mongolia
Taiwan
New_Zealand
Georgia
Germany
Italy
Netherlands
Spain
Russia
0.05

Supplementary Figure 2. Maximum-Likelihood (ML) phylogenetic trees of 8 segments including HA-H1, HA-H3, NA-N1, NA-N2, and internal genes (PB2, PB1, PA, NP, M, and NS). Branches are colored by lineage origin. Branch tips are shaped with circles only for sequences identified in this study. Heatmaps beside the trees indicate the origin of the host and country for each sequence. CS, Classical Swine; EAS, Eurasian Avian-like Swine; A(H1N1)pdm09, 2009 pandemic H1N1; TRIG, Triple Reassortant.
